# The *Hedyotis diffusa* chromosome-level genome and multi-omics analysis provide new insights into the iridoids biosynthetic pathway

**DOI:** 10.3389/fpls.2025.1607226

**Published:** 2025-06-19

**Authors:** Pengyu Chen, Zhuang Huang, Mingzhu Yin, Yu-xin Wen, Qi Jiang, Ping Huang, Rui Qian, Xing Hong, Kaojiang Zhu, Benjiang Xiao, Meng Chen, Shihao Li, Fang Huang, Lin-tao Han

**Affiliations:** ^1^ Hubei Shizhen Laboratory, Wuhan, China; ^2^ Faculty of Pharmacy, Hubei University of Chinese Medicine, Wuhan, China; ^3^ School of Basic Medical Science, Hubei University of Chinese Medicine, Wuhan, China; ^4^ Key Laboratory of Traditional Chinese Medicine Resources and Traditional Chinese Medicine Compound of Ministry of Education, Wuhan, China

**Keywords:** *Hedyotis diffusa*, genome, metabolome, transcriptome, iridoid biosynthetic pathways

## Abstract

**Introduction:**

*Hedyotis diffusa* (Rubiaceae) is a medicinal herb with significant therapeutic potential, primarily attributed to its bioactive iridoid compounds. However, the molecular mechanisms governing iridoid biosynthesis in this species remain poorly characterized, limiting its biotechnological and pharmaceutical applications.

**Methods:**

We generated a telomere-to-telomere (T2T) chromosomal-scale genome assembly of *Hedyotis diffusa* (∼482.30 Mb, anchored to 16 chromosomes) and performed phylogenetic and comparative genomic analyses to investigate its evolutionary history. Additionally, we analyzed the expression patterns of 30 methylerythritol 4-phosphate/mevalonate phosphate (MEP/MVA) pathway genes and 93 iridoid biosynthesis-related genes across different tissues. Gene tree clustering and gene expression analysis were employed to identify candidate genes involved in iridoid post-modification.

**Results:**

The genome assembly revealed a recent species-specific whole-genome duplication (WGD) event in *Hedyotis diffusa*. Expression profiling showed that MEP/MVA pathway genes were predominantly expressed in roots, while iridoid biosynthesis genes exhibited tissue-specific patterns. Three candidate genes—LAMT, OAT, and CYP71—were implicated in iridoid post-modification processes. Gene tree clustering further identified one LAMT gene (*Hd_18862*) and two CYP71D55 homologs (*Hd_18118 and Hd_18119*) as key contributors.

**Discussion:**

This study provides the first T2T genome resource for *Hedyotis diffusa*, elucidating its unique WGD event and evolutionary trajectory. The tissue-specific expression patterns of MEP/MVA and iridoid biosynthesis genes suggest spatial regulation of metabolite production. The identification of LAMT and CYP71D55 homologs advances understanding of iridoid structural diversification. These findings establish a genomic foundation for further exploration of iridoid biosynthesis mechanisms and potential metabolic engineering applications.

## Introduction

**Figure 1:**
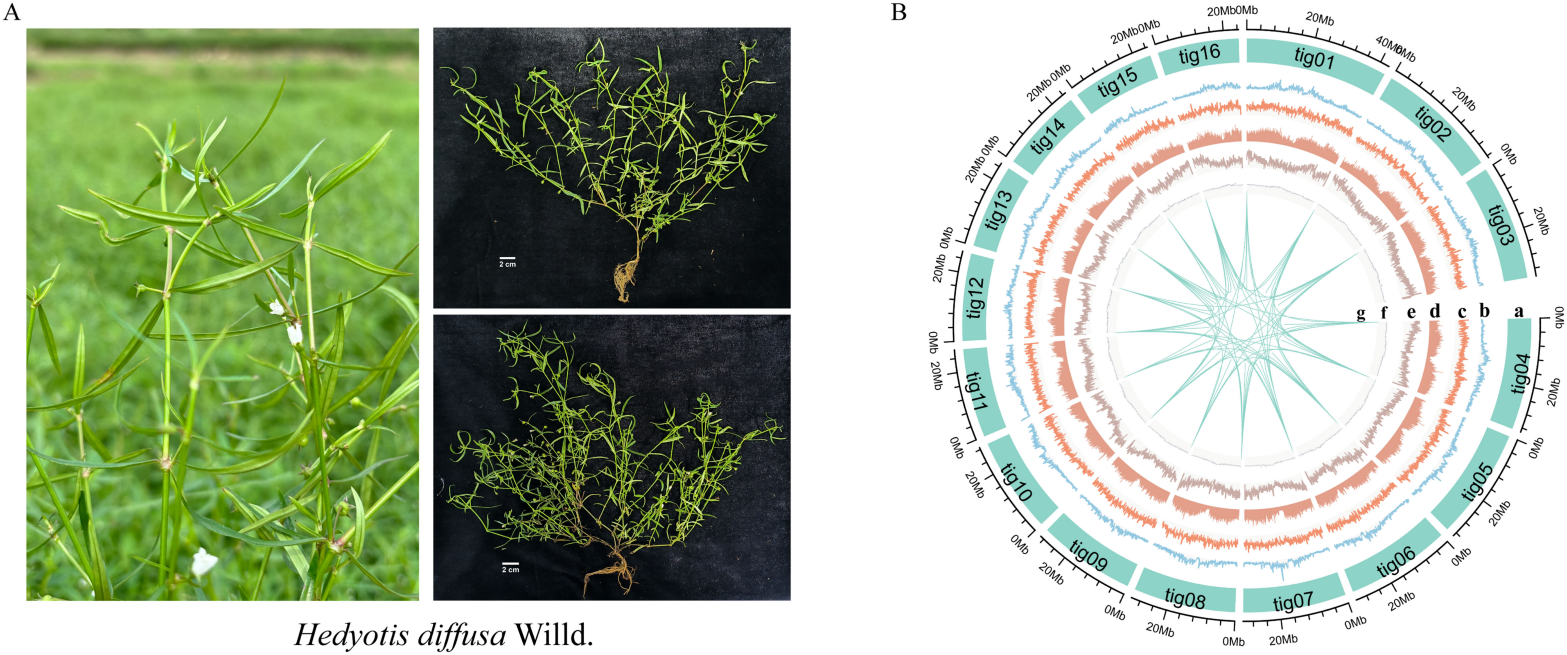
Genomic features of *Hedyotis diffusa*. **(A)** Photos of *Hedyotis diffusa* in nature. **(B)** The distribution of genomic characteristics of *Hedyotis diffusa*. Orbit "a" represents the assembled chromosome. The diameter plots "b-g" represent long terminal repeat (LTR) density, TRF density, repetitive sequence, gene density, GC content and collinearity, respectively. The outermost scale represents the density of genomic features in each 200-kb sliding window on the chromosome.

Rubiaceae is the fourth largest family of angiosperms, comprising approximately 80 genera and over 500 species in China, primarily distributed from the southwest to the southeast regions (Mongrand et al., 2005; Ly et al., 2020). *Hedyotis diffusa* (Hd) is a widely used traditional Chinese medicinal herb with a long history of clinical application (**Figure 1A**). It was first documented in *Shen Nong’s Herbal Classic*, one of the four foundational texts of traditional Chinese medicine (Xiaojuan, 2022). The botanical characteristics and pharmacological properties of Hd have been extensively recorded in authoritative references such as *Chinese Materia Medica*, *Great Dictionary of Chinese Medicine*, and *Flora of China* (Wencai, 2016; Pian, 2017; Editorial department of health newspaper, 2018). According to the *Chinese Pharmacopoeia* (2020 Edition), approximately 30 Chinese patent medicines contain Hd, including Yiganning granules, Shuanghu Qinggan granules, Qingshen granules, and Huahong tablets, among others (Committee NP, 2020). The whole herb of Hd is used medicinally. It is characterized by a bitter, sweet, and cold nature and is associated with the liver, spleen, and stomach meridians. Its primary therapeutic effects include heat-clearing, detoxification, anti-inflammatory, and analgesic properties (Man and Lulong, 2021). Phytochemical studies have identified various bioactive compounds in Hd, including iridoids, flavonoids, anthraquinones, phenolic derivatives, and volatile oils (Chen et al., 2016). Among these, iridoids are particularly significant due to their diverse biological activities, such as antioxidant, anti-inflammatory, neuroprotective, antitumor, and immunomodulatory effects (Lu et al., 2000; Wang et al., 2012; Wang et al., 2019).

Iridoids are commonly found in various plant families such as Rubiaceae, Pyrophoraceae, Cymbiaceae, Scrophulariaceae, Labiatae, and Gentianaceae (Lisheng and Xiangqian, 2009). They belong to a class of cyclopentanopyrane monoterpenes, characterized by two main structures: substituted iridoids and ring opening iridoids (Wang et al., 2020). In Hd, iridoids typically conjugate the C-1 hydroxyl group with glucose to produce iridoid glycosides. The biosynthesis of iridoids involves three stages: precursor formation, iridoids backbone biosynthesis, and preliminary chemical modification processes (Tholl, 2015; Sherden et al., 2018; Rodríguez-López et al., 2022). The biosynthetic pathways for terpenes, including iridoids, share the mevalonate pathway (MVA) and the methylglutarate 4-phosphate pathway (MEP) to produce isopentenyl pyrophosphate (IPP) and dimethylallyl pyrophosphate (DMAPP). Isopentenyl diphosphate delta-isomerase (IDI) facilitates the interconversion of IPP and DMAPP into precursor molecules (Chen et al., 2021). Additionally, DMAPP can be synthesized via the MEP pathway through ISPH (HDR) (Vranová et al., 2012). Generally, the MEP pathway is considered the primary route for synthesizing monoterpenes, including iridoids (Vranová et al., 2012; Chen et al., 2021). Subsequently, the precursor molecules undergo geranyl pyrophosphate synthase (GPPS) catalyzed conversion to geranyl diphosphate. The formation of the Iridoids backbone is facilitated by geraniol synthase (GES), geraniol 8-hydroxylase (G10H), and geraniol 8/10-hydroxylase (8HGO/10HGO) through cyclosynthesis reactions (Kouda and Yakushiji, 2020). Notably, iridoids synthase (IRIS) exhibits stereoselectivity by converting the upstream substrate 8/10 oxogeranial into iridodial and 8-epi-iridodial, leading to the generation of two iridoid biosynthetic pathways (Kries et al., 2017). The biosynthesis of secologanin and catalpol is mediated by a sequential cascade involving iridoids synthase (IRIS) and iridoids oxidase (IO) (Shitiz et al., 2015; Zhang et al., 2019). The secologanin pathway is regulated by a series of enzymes, including 7-deoxyloganetic acid glucosyltransferase (7-DLGT), 7-deoxyloganic acid hydroxylase (7-DLH), loganic acid O-methyltransferase (LAMT), and secologanin synthase (SLS) (Miettinen et al., 2014). In contrast, the catalpol synthesis pathway is governed by multiple enzymes, such as aldehyde dehydrogenase (ALDH), flavanone 3-hydroxylase (F3H), 2-hydroxyisoflavanone dehydratase (2FHD), deacetoxycephalosporin-C hydroxylase (DCH), uroporphyrinogen decarboxylase (UPD), UDP-glucuronic acid decarboxylase (UGD), and squalene monooxygenase (SQM).

Hd contains a diverse array of iridoid metabolites, including deacetyl asperulosic acid, asperuloside, geniposidic acid, and their derivatives (Chen et al., 2016). These iridoids metabolites were also identified in our metabolomic data (**Figure 3B**; **Supplementary Table S13**). Previous studies have elucidated various post modification processes of iridoids, such as glycosylation, methylation, hydroxylation, and isomerization, which ultimately lead to the production of compounds like loganin and catalpol. However, this study focuses on elucidating the biosynthesis of specific key iridoids with bioactive in Hd to better understand the complex pathways underlying plant metabolite diversification. High-quality chromosomal-level genome assembly and gene function annotation provide critical insights into the key regulatory elements of iridoids biosynthesis. Here, we present a telomere-to-telomere (T2T) chromosomal-level genome assembly of Hd, accompanied by comprehensive structural and functional annotation. Phylogenetic and comparative genomic analyses were conducted to determine the evolutionary position of Hd within dicotyledonous plants and to identify a species-specific whole-genome duplication (WGD) event. By integrating genomic, transcriptomic, and metabolomic data, we analyzed the key regulatory genes of the MEP/MVA and iridoid pathways, along with their expression patterns. Furthermore, we predicted post-modification pathway networks based on eight key iridoids and identified three potential regulatory genes. This study not only advances the genomic understanding of Hd but also establishes a foundation for elucidating the biosynthesis mechanisms of iridoids, offering valuable insights for future research and applications.

## Results

### Genome assembly

Utilizing HiFi sequencing technology with rigorous quality control, we generated 33,799,059,475 bp of data (**Supplementary Figure S2**; **Supplementary Table S1**). Comparative analysis of the NT library identified the top 10 species as green plants, confirming no contamination in the sequencing data (**Supplementary Figure S3**, **Supplementary Table S2**). The assembled genome size was 482.30 Mb, with a Contig N50 of 30.97 Mb (**Supplementary Table S3**). Chromosome-level assembly was achieved via Hi-C technology, yielding a 100% chromosome loading rate and pseudomolecular maps for 16 chromosomes. The Hi-C contact map’s lack of significant noise outside the diagonal region indicates high assembly quality (**Figure 1B**; **Supplementary Figure S5**, **Supplementary Table S4**). BUSCO analysis showed 99.29% genome completeness with a high proportion of single-copy sequences (**Supplementary Figure S6**, **Supplementary Table S6**). CEGMA further assessed core gene accuracy and completeness (**Supplementary Figure S7**). GC-depth analysis indicated a GC content of approximately 36.00% and a sequencing depth of 68.39X, affirming the absence of contamination (**Supplementary Figure S8**, **Supplementary Table S7**). These results collectively demonstrate the genome assembly’s high reliability and completeness, especially for conserved genes (**Table 1**).

**Table 1:** Statistics on genome assembly of *Hedyotis diffusa*.

	Items	*Hedyotis diffusa*
Genome assembly		
	genome size (Mb)	482.30
	Contig N50 (Mb)	30.97
	Contigs	16
	Contigs >1,000 kb	16
	Contigs >1,00 kb	16
	Longest contig(bp)	42,645,787
	BUSCO (C)	99.29%
	BUSCO (S)	83.76%
	BUSCO (D)	15.53%
	BUSCO (F)	0.00%
	BUSCO (M)	0.71%
	GC content	0.36
Genome annotation		
	Number of functional genes	37,644
	Number of LTR elements	57,732
	length occupied of LTR(bp)	66,410,398
	Number of SINE elements	7,272
	length occupied of SINE(bp)	1,253,763
	Number of LINE elements	1,439
	length occupied of LINE(bp)	311,031
	Number of Simple repeats(SSR)	60,840
	length occupied of SSR(bp)	1,139,893
	The proportion of repetitive sequences in the genome	40.79%

### Genome annotation and repetitive content

Utilizing biotool software (https://github.com/zxgsy520/biotool), we identified the presence of telomere sequences across all 16 chromosomes. Notably, telomeres were located at both the 5’ and 3’ ends of 14 of these chromosomes. The genome analyzed is characterized as a T2T-sized genome, devoid of chromosomal gaps (**Figure 1B**; **Supplementary Table S8**). The structural annotation of the genome revealed a total of 46,759 genes, with an average gene length of 2,734.57 bp, an average coding sequence (CDS) length of 1,172.84 bp, an average exon length of 253.88 bp, and an average intron length of 431.45 bp. Functional annotation of the protein-coding genes was conducted using various databases, including NR, KEGG, KOG, Swissprot, and GO (**Supplementary Figure S9**, **Supplementary Table S9**). The evaluation results from BUSCO (**Supplementary Table S10**) indicated that 97.88% of gene elements were complete, which serves as an indirect measure of the completeness and reliability of the predicted results. Furthermore, our annotated findings included statistical data on non-coding RNAs (**Supplementary Table S11**). The genome contains 60,840 simple sequence repeats (SSR), which represent 0.24% of the total genomic length. Scattered repeats (transposable elements, TE) constituted 38.17% of the genome, while tandem repeats (TR) and other repeat types accounted for 2.61%. Specifically, long terminal repeats (LTR) comprised 13.77%, DNA transposon factors made up 0.2%, long interspersed nuclear elements (LINE) represented 0.06%, and short interspersed nuclear elements (SINE) accounted for 0.26%. It is noteworthy that the majority of LTRs identified were of the Copia and Gypsy types, which accounted for 8.06% and 5.14%, respectively (**Supplementary Table S12**; **Table 1**).

### Phylogenomics of the Rubiaceae

In this study, we selected 20 dicotyledonous plant species, including Hd, and constructed a phylogenetic tree utilizing 225 single-copy orthologous genes, with Dioscorea alata serving as the outgroup for comparative analysis. Our analysis yielded a total of 30,563 gene families, and by integrating the clustering information of each species’ gene family (**Supplementary Figure S10**, **Supplementary Table S13**), we determined that Hd possesses the highest number of unique gene families, totaling 14,641. Gene Ontology (GO) and Kyoto Encyclopedia of Genes and Genomes (KEGG) enrichment analyses conducted using clusterProfile v3.14.0, revealed that these genes are predominantly enriched in signaling pathways associated with vital regulatory processes, including cell apoptosis, protein digestion and absorption, glycolysis/gluconeogenesis, and RNA degradation. Notably, the enrichment of UDP glycosyltransferase activity (GO: 0008194) and terpene synthesis activity (GO: 0010333) underscores their significant role in the biosynthesis of iridoids and their glycosides in Hd (**Supplementary Figures S12A, B**). Furthermore, Hd exhibited a substantial expansion of gene families, comprising 3,348 expansion genes and 1,132 contraction genes (**Supplementary Figure S11**). Functional annotation indicated that the expanded gene families were particularly rich in Gene Ontology (GO) terms. Specifically, the entries for chitinase activity (GO: 0004568), chitin catalytic process (GO: 0006032), and chitin binding (GO: 0008061) are all associated with the function of chitinase. Chitinase is capable of hydrolyzing chitin, a key component of the cell walls of plant pathogenic fungi, thereby exhibiting antibacterial properties and directly eliminating pathogenic fungi while also triggering plant defense mechanisms (Vaghela et al., 2022). Additionally, UDP glycosyltransferase activity (GO: 0008194) and acetyltransferase activity (GO: 0016747) facilitate the post-modification processes of iridoids (**Supplementary Figure S12C**).

### Whole-genome duplication

**Figure 2:**
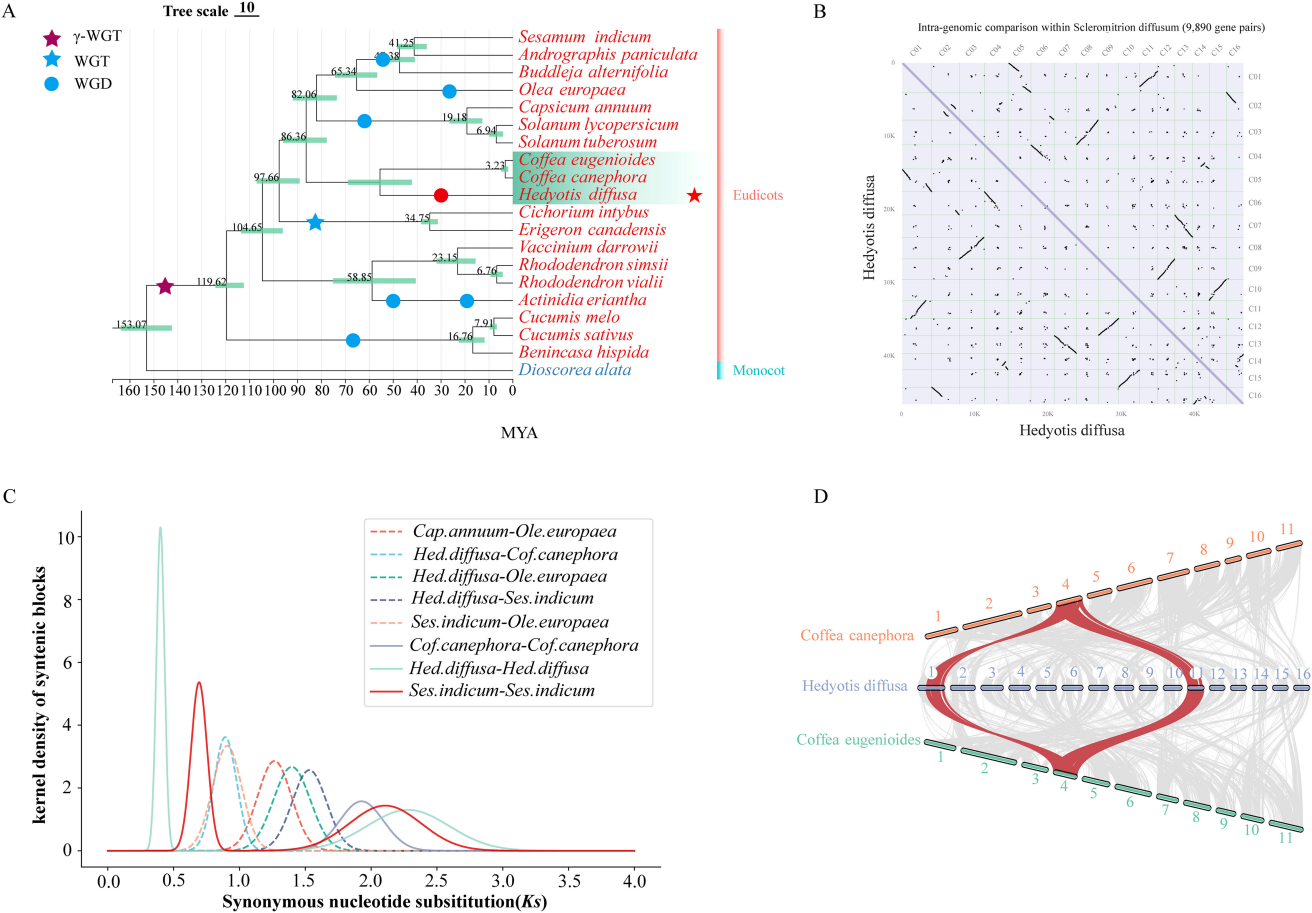
Phylogenetic and Comparative Genomic Studies. **(A)** Phylogenetic analysis of dicotyledonous plants with *Hedyotis diffusa* and other known genomic information. The evolutionary tree annotates the whole genome replication (WGD) events of known species and the estimated divergence time of each node; **(B)** Dotplot of synteny blocks within the *Hedyotis diffusa* genome, C01-C16 represents a grid of 16 chromosomes; **(C)** Distribution map of synonymous substitution rates (Ks) between *Hedyotis diffusa* and four other dicotyledonous species homologous gene pairs **(D)** The collinearity graph of Coffea and *Hedyotis diffusa*.

The most recent whole genome duplication (WGD) event in Hd has been identified through phylogenetic tree and collinearity analyses, in conjunction with previously documented WGD events in various species (**Figure 2A**) (Yu et al., 2017; Xie et al., 2019; Wang et al., 2021; Fan et al., 2022; Wu et al., 2022; Yao et al., 2022). The core eudicotyledonous common gamma whole genome triplication (γWGT) event is estimated to have occurred approximately 150 million years ago (MYA), with a peak synonymous substitution rate (Ks) of around 2.0. The Ks curve indicates that the peak Ks value (0.4) for Hd was observed subsequent to its divergence from *Coffea canephora*, and later than the WGD event in *Sesamum indicum*, which occurred approximately 48 MYA. Furthermore, the Ks peak for *Olea europaea* (approximately 0.3) corresponds to a WGD event dated to about 25 MYA, which is marginally more recent than that of Hd (**Figure 2C**; **Supplementary Figure S13**). The WGD time for Hd has been calculated using the formula: *T=Ks/2r*, yielding an estimate of approximately 32 MYA. The presence of multiple homologous gene pairs within the genomic structure of Hd substantiates the occurrence of recent WGD events in this species (**Figure 2B**). Notably, there has been no subsequent WGD event in *Coffea canephora* and *Coffea eugenioides* following the gamma WGT event. Consequently, a classical collinearity model further elucidates the 1:2 relationship between the genomic regions of *Coffea* and Hd (**Figure 2D**).

**Figure 3:**
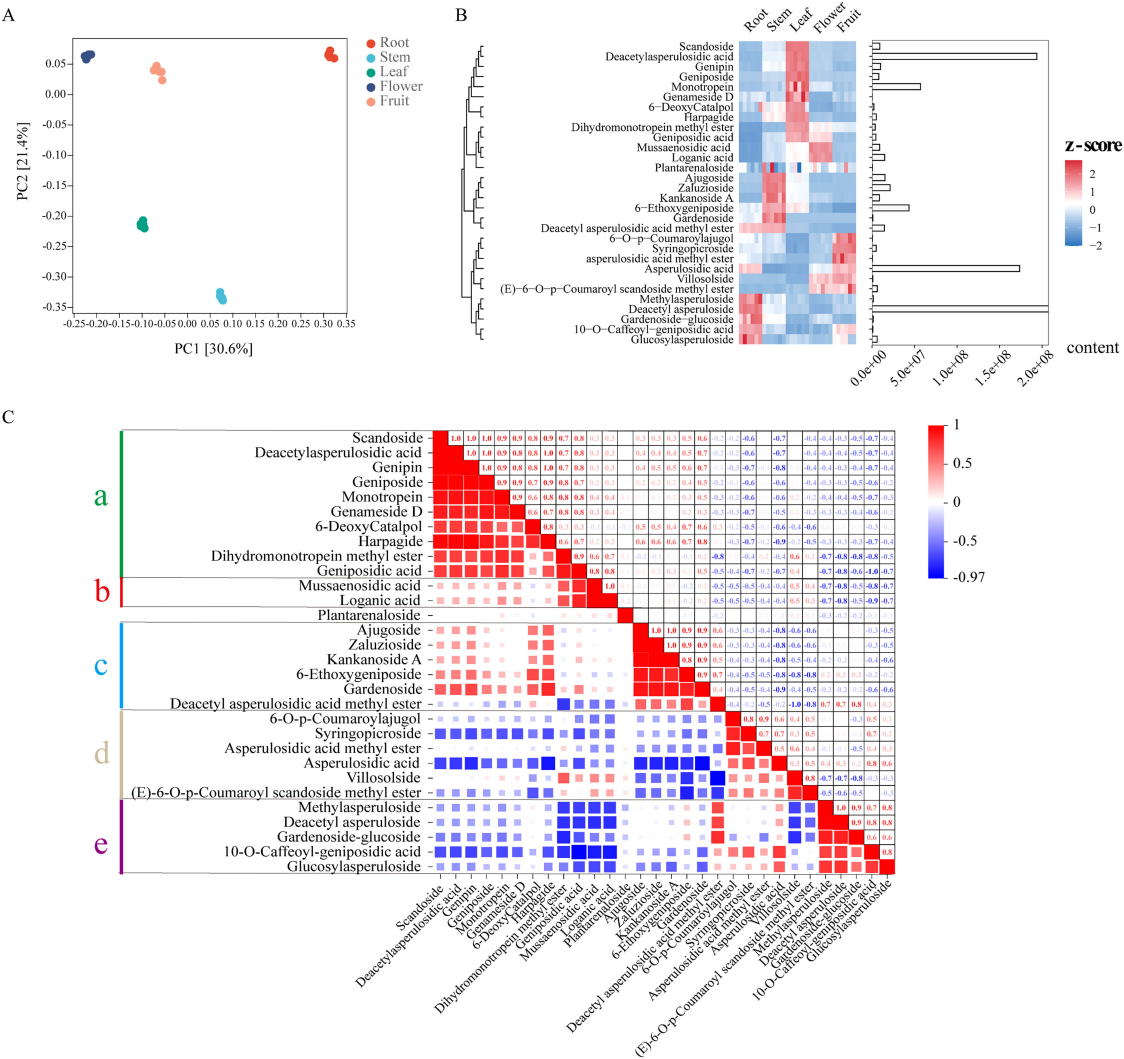
The distribution of iridoid in *Hedyotis diffusa* in 5 plant tissues (Root, Stem, Leaf, Flower, Fruit) was analyzed by metabolomics. **(A)** PCA principal component analysis. **(B)** Cluster heat map analysis of 30 iridoid metabolites, the relative content of metabolites was standardized by z-score. The redder the block color of the heat map, the more positively enriched the metabolites in the corresponding tissues, while the bluer the block color, the more negatively enriched the metabolites in the corresponding tissues. The bar chart shows the total relative content of metabolites, with the horizontal axis representing the relative content values. **(C)** Analysis of the intrinsic correlation of 30 metabolites, a, b, c, d and e represent the five correlation cluster blocks.

### Metabolomics analysis of iridoids in Hd

In this study, we conducted extensive targeted metabolomics analyses utilizing the roots, stems, leaves, flowers, and fruits of Hd. The results of principal component analysis (PCA) indicated a high degree of consistency among the five biological replicates within each group, while also demonstrating clear differentiation among the five tissues based on the conditions of PC1 (30.6%) and PC2 (21.4%) (**Figure 3A**). We identified 30 iridoid metabolites classified as Class II monoterpenoids (**Supplementary Table S13**) and elucidated their relative concentrations and tissue distribution through hierarchical clustering heat maps (**Figure 3B**). The findings revealed that these 30 iridoids were broadly distributed across various tissues, with 10 iridoids specifically accumulating in leaves, while only Mussaenosidic acid and Loganic acid exhibited significant accumulation in flowers. The three most abundant iridoids–Deacetyl asperuloside, Deacetyl asperulosidic acid, and Asperulosidic acid –were found to be highly concentrated in the roots, leaves, and fruits, respectively. **Figure 3** illustrates the intrinsic correlations among these 30 iridoids, which can be categorized into five distinct clusters based on their correlation (a, b, c, d, e). In fact, the correlation cluster aligns with the tissue distribution of the iridoid metabolites; cluster a (leaf accumulation) is positively correlated with cluster b (flower accumulation) and cluster c (stem accumulation), with the exception of Deacetyl asperulosidic acid methyl ester (**Figures 3B, C**). Conversely, these clusters exhibit negative correlations with clusters e and f.

### KEGG and GO enrichment analysis of differential genes

**Figure 4:**
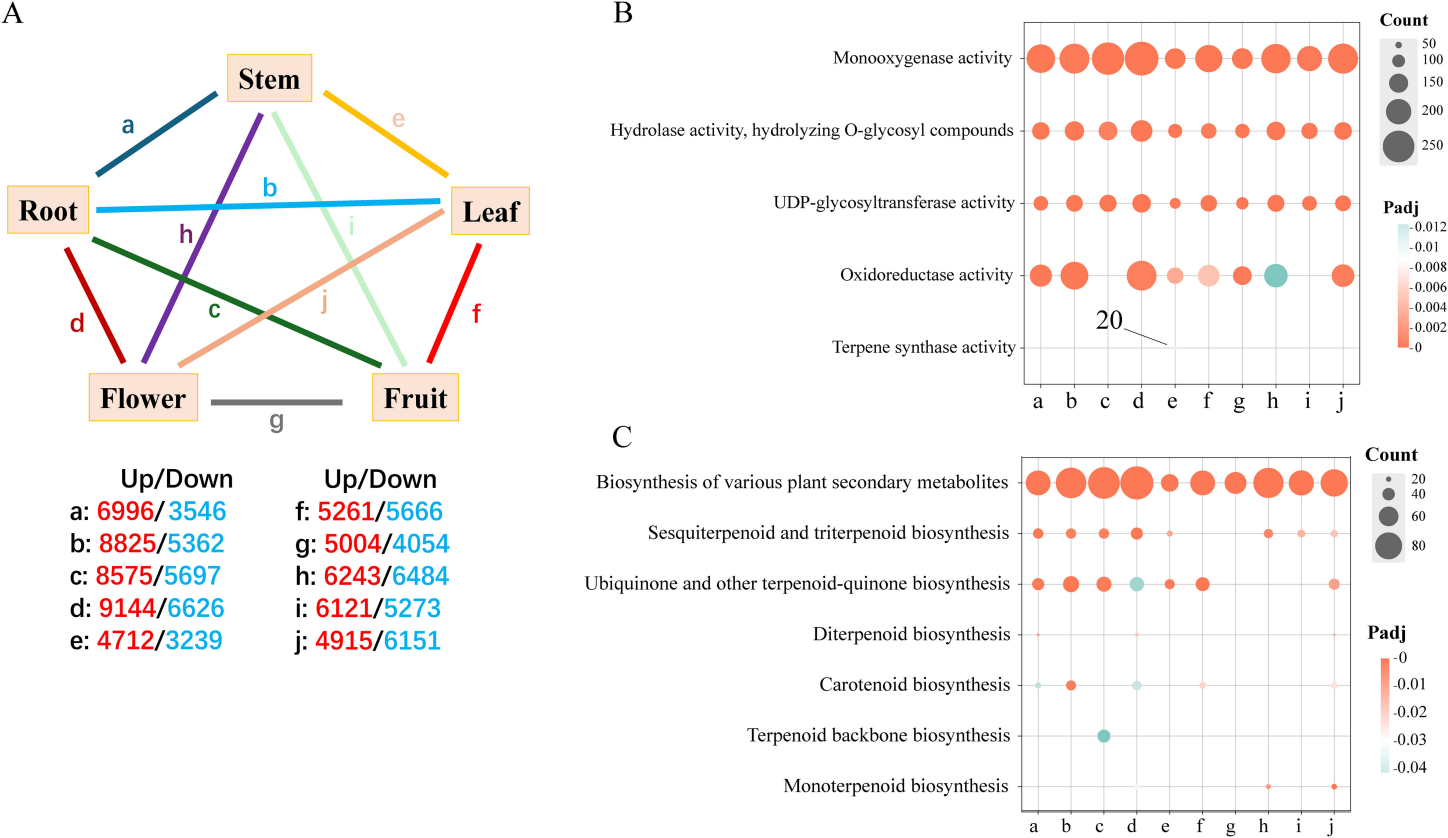
GO and KEGG enrichment analysis. **(A)** DEG group comparison of pentagram charts. This section covers the analysis of pairwise comparative combinations of 5 tissues: a: Root and Stem; b: Root and Leaf; c: Fruit and Root; d: Flower and Root; e: Stem and Leaf; f: Leaf and Fruit; g: Flowers and Fruit; h: Flower and Stem; i: Stem and Fruit; j: Flower and Leaf. The red and blue numbers indicate the number of DEGs up-regulated and down-regulated, respectively. **(B, C)** Bubble maps were used for GO and KEGG enrichment analysis of up-regulated genes.

In this investigation, transcriptome sequencing was performed on root, leaf, flower, and fruit tissues of Hd, utilizing six biological replicates for each tissue type. The Q30 quality score for each sample exceeded 92%, with GC content varying between 41.76% and 46.23%, yielding an average of approximately 44.53% (**Table 2**). To comprehensively elucidate the functions of differentially expressed genes, we conducted Gene Ontology (GO) and Kyoto Encyclopedia of Genes and Genomes (KEGG) enrichment analyses by establishing a series of tissue comparison combinations: a) Root and Stem; b) Root and Leaf; c) Fruit and Root; d) Flower and Root; e) Stem and Leaf; f) Leaf and Fruit; g) Flowers and Fruit; h) Flower and Stem; i) Stem and Fruit; j) Flower and Leaf. The analysis identified the number of up-regulated and down-regulated genes within each comparison group (**Figure 4A**). The functional enrichment outcomes from the GO and KEGG analyses highlighted associations with terpenoid biosynthesis. In the GO analysis of up-regulated genes, the differentially expressed genes across each comparison group were found to be involved in monooxygenase activity, hydrolase activity (specifically hydrolyzing O-glycosyl compounds), and UDP-glycosyltransferase activity. Notably, only 20 differentially expressed genes in comparison group e were enriched for terpene synthase activity, which may correlate with the accumulation of iridoids in the leaves (**Figure 4B**). The KEGG analysis of up-regulated genes revealed that all comparison combinations were enriched in the biosynthesis of various plant secondary metabolites, with comparison group c primarily enriched in terpenoid backbone biosynthesis, while groups d, h, and j were predominantly focused on monoterpenoid biosynthesis. Additionally, several comparison groups also emphasized other terpenoid-related pathways, including sesquiterpenoid and triterpenoid biosynthesis, ubiquinone and other terpenoid-quinone biosynthesis, as well as carotenoid biosynthesis (**Figure 4C**). These findings indicate that multiple genes in Hd are involved in catalyzing the biosynthesis of monoterpenes and other terpenes through diverse pathways, with the biosynthesis of monoterpenes and their precursors primarily localized in the roots, stems, and leaves.

**Table 2:** Quality evaluation of transcriptome sequencing in *Hedyotis diffusa*.

Sample	Clean reads(bp)	Clean bases (bp)	Q30 rate(%)	GC(%)
Root1	89,107,874	12,756,337,230	94.74	41.76
Root2	65,932,790	9,850,514,486	92.64	44.52
Root3	59,591,558	8,896,523,212	93.21	44.66
Root4	58,963,998	8,811,128,814	92.37	44.40
Root5	68,559,600	10,242,596,688	92.76	44.70
Root6	50,233,250	7,508,530,554	92.82	44.36
Stem1	51,344,284	7,677,441,346	93.41	43.87
Stem2	58,105,430	8,681,408,544	92.58	43.75
Stem3	57,978,376	8,652,113,572	92.52	43.84
Stem4	60,857,522	9,031,134,028	93.95	45.05
Stem5	35,561,314	5,302,516,944	93.48	45.20
Stem6	50,173,542	7,466,745,600	93.16	45.94
Leaf1	55,646,876	8,309,534,368	93.07	44.47
Leaf2	70,883,230	10,567,267,298	94.02	43.93
Leaf3	65,193,334	9,718,086,748	93.39	44.43
Leaf4	57,180,448	8,535,391,710	92.88	44.56
Leaf5	63,418,508	9,477,921,558	92.96	45.23
Leaf6	63,053,164	9,405,909,330	92.96	44.12
Flower1	54,095,746	8,073,571,620	92.53	44.33
Flower12	58,849,426	8,787,155,302	92.83	43.43
Flower13	57,538,102	8,595,297,310	92.65	44.40
Flower14	95,722,126	13,960,706,688	96.30	43.36
Flower15	47,771,764	7,141,134,140	93.02	45.17
Flower16	51,855,550	7,755,249,354	92.89	44.63
Fruit1	52,489,174	7,843,249,626	92.56	45.22
Fruit2	49,423,942	7,376,752,918	92.38	45.20
Fruit3	56,914,132	8,498,693,528	92.78	45.15
Fruit4	64,722,940	9,665,449,566	92.81	44.91
Fruit5	54,217,486	8,100,853,794	93.82	45.10
Fruit6	104,223,352	15,432,761,858	94.15	46.23

### Expression patterns of iridoid biosynthetic pathway genes in different tissues

**Figure 5:**
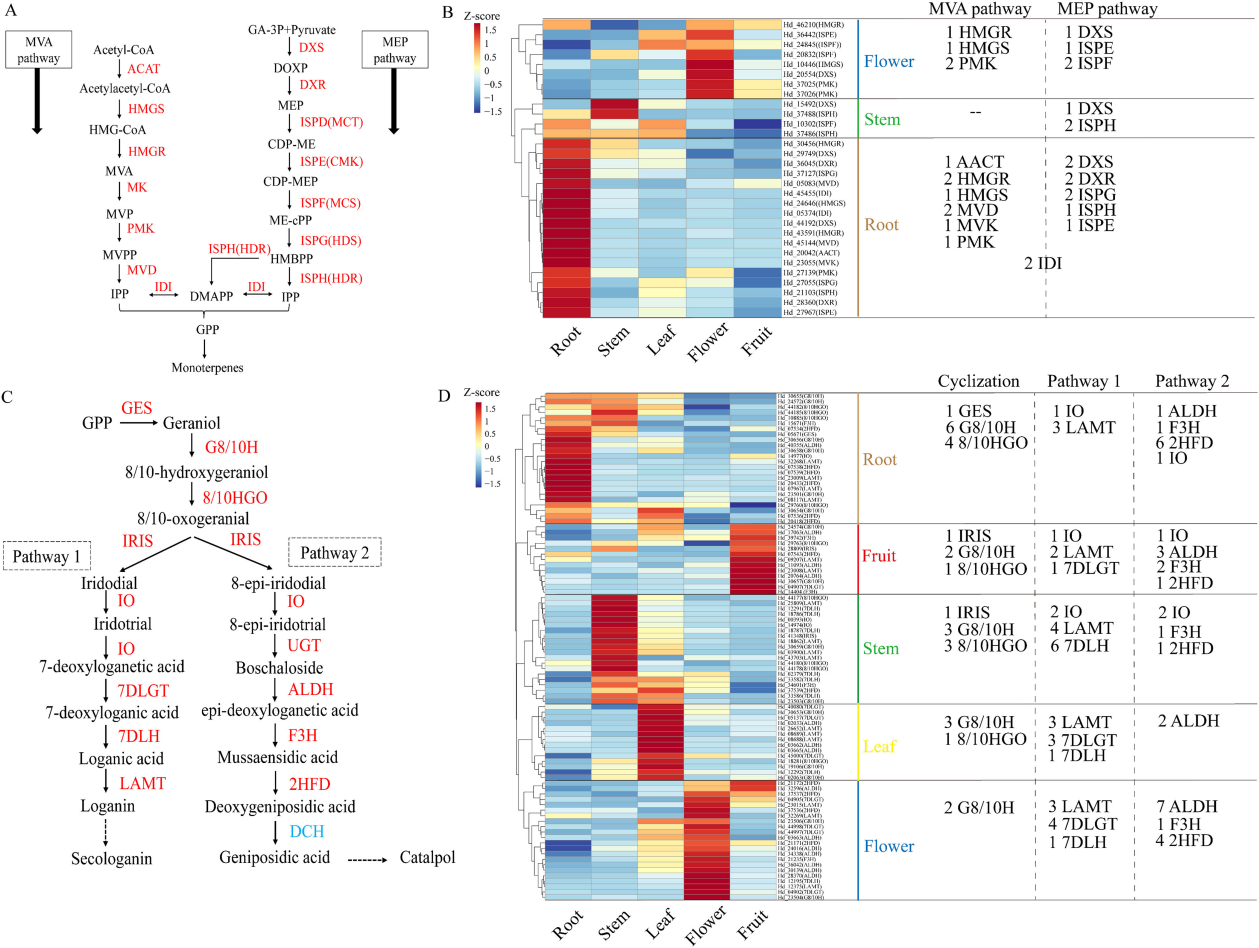
Analysis of gene expression patterns of iridoid biosynthetic pathways. **(A)** MVA and MEP pathways. **(B)** Heat map of regulatory gene analysis of MVA and MEP pathways. **(C)** iridoid biosynthesis pathways, including cyclization process of backbone and preliminary chemical modification process (pathway 1 and pathway 2). **(D)** Heat maps of regulatory genes of two iridoid biosynthetic pathways.

Previous research has demonstrated that the MVA and MEP pathways regulate the production of iridoid precursors, with terpenoids utilizing these pathways to synthesize IPP and subsequently activate the monoterpene-iridoid biosynthesis pathway via GPP (Geranyl pyrophosphate) synthesis (Bohlmann et al., 1998; Ye et al., 2019). In this study, 30 genes associated with the MVA/MEP pathways were identified, including AACT(1), HMGS(2), HMGR(3), MVK(1), PMK(3), MVD(2), and MEP pathway genes DXS(4), DXR(2), ISPE(2), ISPF(2), ISPG(2), ISPH(3), along with two IDI genes (**Figure 5A**). Heat maps revealed that these genes were predominantly expressed in roots, leaves, and flowers. In flowers, genes associated with the MVA pathway (1 HMGR, 1 HMGS, 2 PMK) and the MEP pathway (1 DXS, 1 ISPE, 1 ISPF) were highly expressed. The stem showed a predominance of the MEP pathway, including 1 DXS and 1 ISPH. Notably, the root exhibited high expression of both MVA pathway genes (1 AACT, 2 HMGR, 1 HMGS, 2 MVD, 1 MVK, 1 PMK) and MEP pathway genes (2 DXS, 2 DXR, 2 ISPG, 1 ISPH, 1 ISPE), along with 2 IDI genes. These findings suggest that the root is a crucial site for the formation of terpenoid precursors via the MVA/MEP pathways in Hd, facilitating the accumulation of synthetic raw materials. Despite the low gene enrichment in leaves and fruits, we observed high expression of 12 MEP pathway genes in leaves and 4 MVA pathway genes in fruits. These findings suggest that the MEP pathway is predominantly active in stems and leaves, whereas the MVA pathway is active in fruits (**Figure 5B**).

The iridoid biosynthetic pathway involves the cyclization process of the iridoid backbone, catalyzed by IRIS, which converts GPP into iridodial and 8-epi-iridodial. This process is regulated by the expression of 28 genes encoding key enzymes, including GES, G8/10H, 8/10HGO, and IRIS. Additionally, a total of 65 coding genes for enzymes in two distinct iridoid pathways were identified: Pathway 1, involving IO, 7DLGT, 7DLH, and LAMT, and Pathway 2, involving UGT, ALDH, F3H, and 2HFD (**Figure 5C**). A heat map of gene expression revealed that genes involved in iridoids biosynthesis are widely distributed across five tissues, with cyclization genes predominantly expressed in roots and stems. Notably, multiple G8/10H and 8/10HGO genes were enriched in all tissues, underscoring their role in regulating iridoids backbone synthesis. Additionally, Pathway 1 genes were more highly expressed in stems, whereas Pathway 2 genes were more prominent in flowers. This widespread gene enrichment contributes to the tissue-specific distribution of iridoid metabolites in Hd. However, our transcriptome data did not detect DCH in Pathway 2, suggesting that geniposidic acid in metabolites is not synthesized via Pathway 2 but may interact with Pathway 1 (**Figure 5D**).

### Validation of encoding genes relation to metabolism of iridoids by qRT-PCR

**Figure 6:**
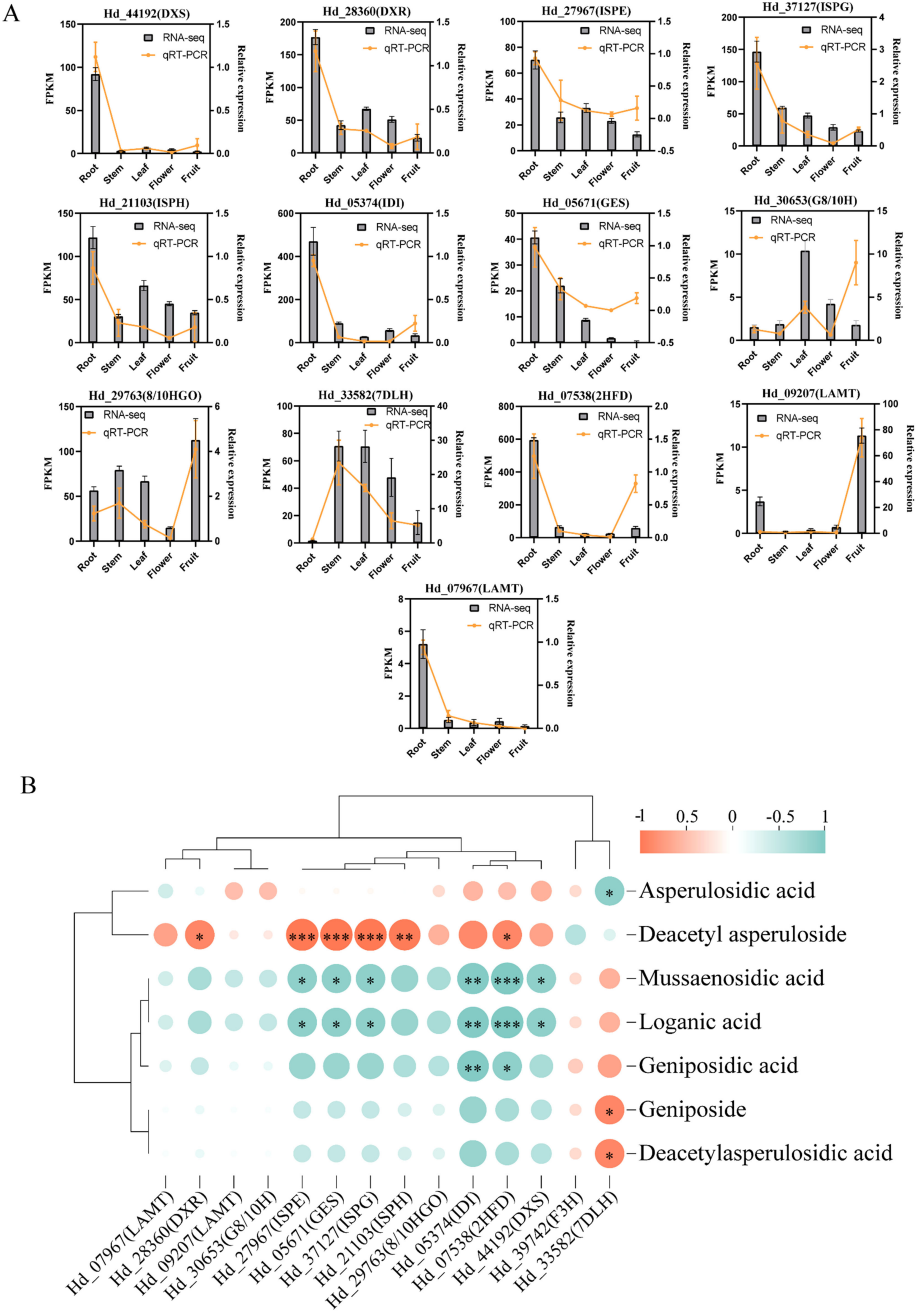
Transcript of *Hedyotis diffusa* and gene expression from 5 tissues verified by qRT-PCR. Roots, stems, leaves, flowers, fruits. **(A)** Gene validation results of qRT-PCR and overlay analysis with transcriptomic data. **(B)** Correlation analysis was performed between the relative expression levels of qRT-PCR and eight compounds associated with the biosynthesis of several high-content iridoids. Orange-red and turquoise represent positive and negative correlations. The size and color depth of the circles are employed to represent the level of importance, *P < 0.05; **P < 0.01; *P < 0.001.

To validate the reliability and authenticity of transcriptome data, we conducted qRT-PCR analysis to confirm the expression of key genes involved in the iridoid biosynthesis pathways across roots, stems, leaves, flowers, and fruits. We selected 13 coding genes for qRT-PCR validation, primarily from the MEP pathway, the cyclization process, and pathway1. The results indicated that most gene expression patterns aligned with the transcript data, except for *Hd_27967* (ISPE), *Hd_21103* (ISPH), and *Hd_30653* (G8/10H) (**Figure 6A**). Specifically, MEP pathway genes *Hd_44192* (DXS), *Hd_28360* (DXR), and *Hd_37217* (ISPG) exhibited high expression in roots. Cyclization process genes *Hd_05671* (GES) and *Hd_29763* (8/10HGO) were highly expressed in roots and fruits, respectively. Additionally, the two LAMT-encoding genes, *Hd_09207* (LAMT) and *Hd_07967* (LAMT), showed distinct expression patterns, with high expression in fruit and root, respectively (**Figure 6A**).

We selected eight iridoids, based on three with high relative content, that share similar chemical structures and biosynthetic pathways. We then examined the correlation between the relative content of these iridoid metabolites and the expression levels of enzyme-coding genes using qRT-PCR (**Figure 6B**). Significant positive correlations were found in eight pairs, while 15 pairs showed significant negative correlations. Deacetyl asperloside, the most abundant iridoids, was significantly positively correlated with the genes DXR, ISPE, ISPG, ISPH, and 2HFD (*P*<0.05). These genes were negatively correlated with mussaenosidic acid, loganic acid, geniposidic acid, geniposide, and deacetyl asperulosidic acid. Additionally, asperulosidic acid was significantly negatively correlated with 7DLH (*P*<0.05). These genes collectively regulate iridoids biosynthesis through multiple coordinated interactions (**Figure 6B**).

### Gene prediction for regulating key iridoids postmodification

**Figure 7:**
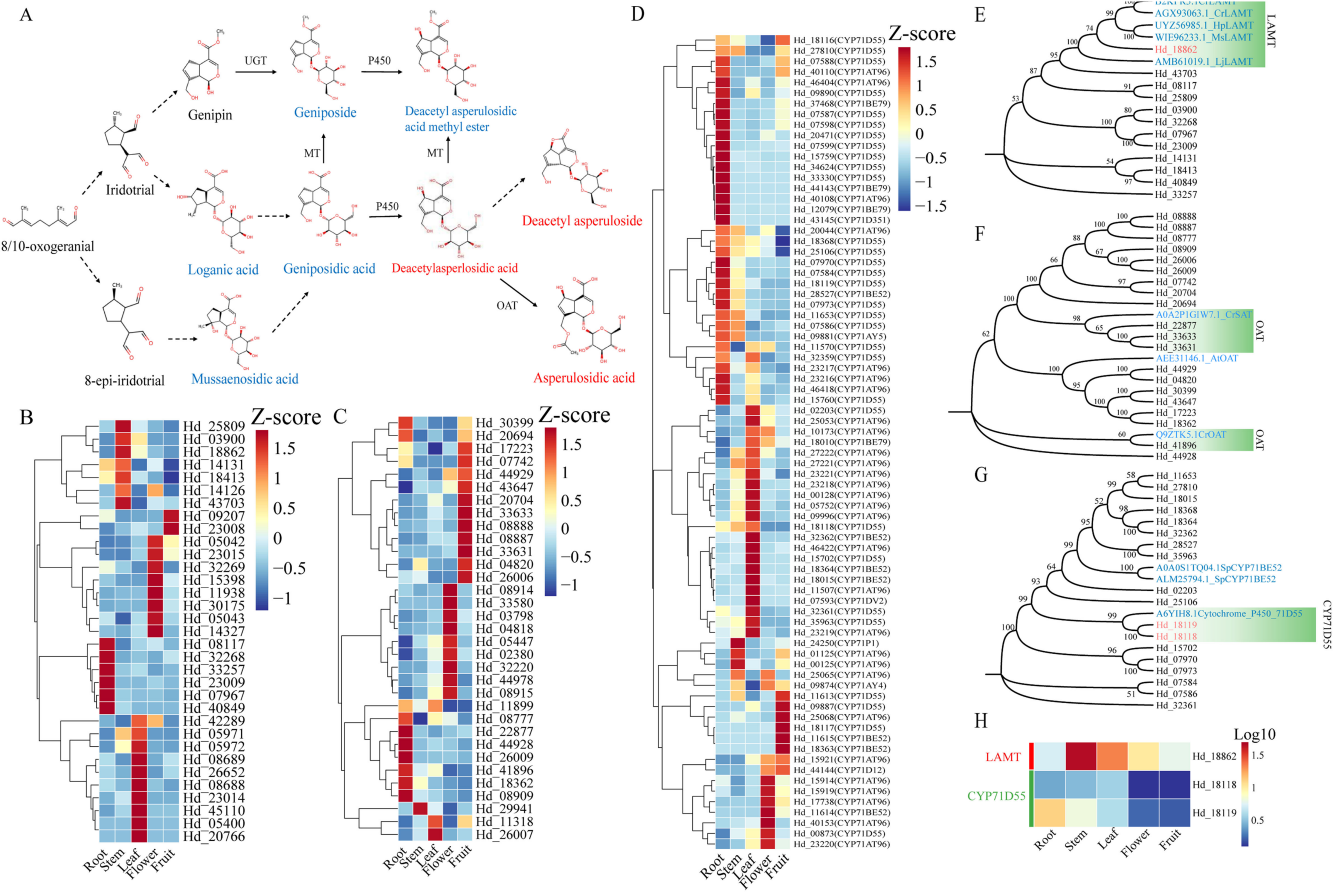
The hierarchical clustering heat map combined with gene tree predicted the key regulatory genes of the post-modification process of iridoid. **(A)** Structural diagram of postbiosynthetic modification pathway inferred by high content of iridoid and related compounds; **(B)** Heat map of gene expression pattern of LAMT (Loganic acid O-methyltransferase); **(C)** Heat map of gene expression pattern of OAT(O-acetyltransferase); **(D)** Heat map of gene expression pattern of CYP71 gene family; Increased and decreased gene expression were identified using dark red and dark blue color blocks, respectively. **(E-G)** The gene tree of LAMT, OAT, and CYP71 enzyme-coding genes was constructed using the Neighbor-Joining method. **(H)** Heat maps of coding genes for 3 kinds of enzymes (data scaled with Log10).

We investigated the post-modification processes of eight iridoid metabolites in Hd, focusing on pathways involving methyltransferase (MT), O-acetyltransferase (OAT), and Cytochrome P450s (**Figure 7A**). Loganic acid O-methyltransferase (LAMT), a key methyltransferase, typically methylates the -OH group of the iridoid C-4 carboxyl group, as corroborated by several studies (Petronikolou et al., 2018; Kang et al., 2021; Hao et al., 2023). MT requires two kinds of coding genes with varying expression patterns for the biosynthesis of Geniposide and Deacetyl asperulosidic acid methyl ester. We identified 17 genes annotated as LAMT in the transcriptome that exhibit expression patterns similar to those of Geniposide and Deacetyl asperulosidic acid methyl ester (**Figure 7B**; **Supplementary Table S14**). Additionally, we identified 20 OAT coding genes consistent with the enrichment pattern of Asperulosidic acid, the catalytic product of OAT (**Figure 7C**; **Supplementary Table S14**). The CYP450 gene family plays a crucial role in terpenoid biosynthesis, with the CYP71 family specifically implicated in monoterpenoid biosynthesis through hydroxylation of specific metabolites (Drew et al., 2013; Yuting et al., 2020). Our comprehensive analysis of the CYP71 gene cluster in Hd revealed that this family includes two key enzymes, CYP71D55 and CYP71AT96, with notable gene coding activity. Heat map analysis indicated that CYP71D55 is predominantly expressed in the root, while CYP71AT96 is expressed across various tissues (**Figure 7D**). Additionally, CYP71BE52 and CYP71BE79 exhibit high expression in the root, leaf, and fruit, respectively. Notably, despite multiple coding genes, there is no evidence supporting that CYP71AT96 is involved in hydroxylation regulation. Consequently, excluding CYP71AT96, we identified 18 genes with expression patterns similar to Deacetyl asperulosidic acid methyl ester and Deacetyl asperulosidic acid, corresponding to two enzyme types: CYP71D55 and CYP71BE52 (**Supplementary Table S14**).

To validate the gene’s function, we integrated the screened gene with known functional enzymes (LAMT, OAT, CYP71D55, and CYP71BE52) from other plants into a bootstrap consensus gene tree using the Neighbor-Joining method (Felsenstein, 1985; Saitou and Nei, 1987). Sequence similarity analysis revealed that among the LAMT candidate coding genes, only *Hd_18862* showed homology with CrLAMT (B2KPR3.1/AGX93063.1), HpLAMT (UYZ56985.1), and MsLAMT (WIE96233.1), with homologies of 75.75%, 78.21%, and 78.81%, respectively (**Figure 7E**). For OAT candidate genes, *Hd_41896* clustered with CrOAT (Q9ZTK5.1), while *Hd_22877*, *Hd_33633*, and *Hd_33631* clustered with CrSAT (A0A2P1GIW7.1), showing homologies of 32.76%, 43.09%, 46.31%, and 48.18%, respectively (**Figure 7F**). Within the CYP71 family, only Hd_18118 and Hd_18119 clustered with CYP71D55 (A6YIh8.7), with homologies of 61.89% and 62.11%, respectively (**Figure 7G**). These results suggest that, except for OAT, the genes exhibit high homology with known amino acid sequences, indicating potential functional consistency. We identified three genes, including one LAMT (*Hd_18862*) and two CYP71D55 (*Hd_18118* and *Hd_18119*), which display distinct expression patterns (**Figure 7H**).

## Discussion

The clinical and research significance of Hd in Chinese herbal medicine underscores the value of its high-quality chromosome-scale genome assembly. We have developed a telomere-to-telomere (T2T) genome assembly with a size of 482.30 Mb, utilizing PacBio sequencing and Hi-C assisted techniques. Our phylogenetic analysis elucidates the evolutionary placement of Hd within dicotyledonous plants. As plant genome research advances, the phylogenetic relationships among dicotyledonous groups are becoming increasingly clear. Among the three Rubiaceae species studied, Hd, Coffea canephora, and Coffea eugenioides, the latter two are closely related, with Coffea differentiating more recently following its divergence from Hd (**Figure 3A**). Current evidence indicates that the Coffea genus is not undergoing new whole-genome duplication (WGD) events (Wang et al., 2021; Salojärvi et al., 2024). Through Ks curves, collinearity analysis, and comparisons with known WGD species, we confirmed a unique WGD event in Hd (Yu et al., 2017; Fan et al., 2022; Qi et al., 2022; Yao et al., 2022). We estimate the WGD event occurred approximately 32 MYA (**Figure 3A**). The recent whole-genome duplication (WGD) event is considered a significant contributor to the abundance of unique gene families in Hd (**Supplementary Figure S10**). KEGG and GO analyses indicate that these gene families are linked to various entries in terpenoid biosynthesis (**Supplementary Figures S12A, B**). Genomic analysis reveals that following the WGD event, there was a substantial expansion of gene families within the Hd genome, a member of the Rubiaceae family (**Supplementary Figure S11**). GO functional enrichment analysis demonstrates that these expanded gene families are implicated not only in plant defense but also in the synthesis of iridoid glycosides, particularly through UDP-glycosyltransferase activity (**Supplementary Figure S12C**). It is posited that Hd has developed the capacity to adapt to environmental changes and to enhance biosynthetic diversity throughout its evolutionary history. Notably, in various plant tissues, enzymes involved in the iridoid synthesis pathway are encoded by multiple genes. This redundancy resulting from gene duplication may facilitate the regulation and optimization of these biosynthetic pathways under diverse tissue types and environmental conditions, thereby augmenting the metabolic diversity of the plant.

In this study, we employed targeted metabolomics to analyze iridoid metabolites across five tissues of Hd, identifying 30 iridoids. Among these, deacetyl asperuloside, deacetyl asperulosidic acid, and asperulosidic acid were most abundant. Asperulosidic acid notably inhibited NO production in LPS-stimulated RAW264.7 cells, with an IC50 of 5.75 ± 0.85 μM (Tran et al., 2019). Both asperulosidic acid and deacetyl asperulosidic acid reduced NO, PGE_2_, and TNF-α production in these cells and inhibited NF-κB and MAPK pathways, underscoring their anti-inflammatory potential (He et al., 2018). These iridoids, despite their structural similarity, displayed distinct tissue distribution, suggesting a spatiotemporal transport mechanism for metabolites. We propose that these iridoid metabolites are crucial to the anti-inflammatory effects of Hd, as evidenced by their tissue-specific enrichment patterns. Furthermore, certain genipin derivatives, including geniposidic acid, geniposide, scandoside, monotropein, and deacetyl asperulosidic acid, are co-enriched in the leaves. This specific tissue distribution indicates that the leaves may serve as a crucial site for the biosynthesis of compounds like deacetyl asperulosidic acid in Hd (**Figure 1B**).

We examined the expression of iridoid biosynthetic pathway genes using transcriptomic data. Previous studies have indicated that monoterpenoids are primarily synthesized via the MEP pathway (Vranová et al., 2012; Vranová et al., 2013; Opitz et al., 2014). Our findings confirm that while the MEP pathway is prevalent across five tissues and plays a significant regulatory role in stems and leaves, the MVP pathway also contributes to iridoids biosynthesis in roots alongside the MEP pathway. Notably, genes encoding the MEP/MVA pathways are highly expressed in roots, aligning with the expression patterns of cyclization genes (GES, G8/10H, and 8/10HGO). This observation, supported by earlier research (Kang et al., 2022), suggests a co-regulatory mechanism in root iridoids formation involving cyclization genes (**Figures 1B**, **4D**). Genes involved in both iridoid biosynthesis pathways are broadly distributed across tissues, lacking specificity, which implies that iridoid metabolites production in Hd exhibits spatiotemporal transport characteristics. The pathway from iridodial to secologanin is now well-characterized (Kouda and Yakushiji, 2020). Combining chemical structure and tissue distribution patterns, we suggest that loganic acid in pathway 1 may further lead to the biosynthesis of geniposidic acid and its derivatives.

We performed a correlation analysis between the relative expression of genes in the MEP/MVA pathway and the iridoid biosynthesis pathway, along with the relative contents of 30 iridoids (**Supplementary Figure S13**). The findings revealed that the MEP pathway exhibited stronger positive correlations than the MVA pathway, suggesting its more significant role in synthesizing iridoids precursors. Notably, more genes in the MEP/MVA pathway were strongly correlated with iridoids found in roots, such as methylasperuloside, deacetyl asperuloside, and deacetyl asperulosidic acid methyl ester, aligning with prior analyses. Furthermore, genes coding for iridoids cyclization showed strong correlations with iridoids in leaves and stems. Additionally, genes encoding pathway 1 and pathway 2 demonstrated a similar extent of positive correlation with iridoids, indicating their combined influence on different iridoids.

Although the research on the biosynthetic pathways of iridoids precursors and backbones has been relatively mature, the understanding of the specific post modification processes of iridoids is still limited. Therefore, this study predicted a post modification pathway involving eight major iridoids (**Figure 7A**). Specifically, this pathway involves three types of enzymes: O-methyltransferase (MT), O-acetyltransferase (OAT), and cytochrome P450s (P450). Loganic acid O-methyltransferase (LAMT) plays a role in secologanin biosynthesis within the pathway1 pathway. We deem that LAMT catalyzes the O-methylation of iridoids at C-11, potentially enabling the production of geniposide and deacetyl asperulosidic acid methyl ester (Murata et al., 2008). Cluster analysis of LAMT candidate genes (**Figure 7E**) revealed that only *Hd_18862* clusters with known LAMT coding genes. Notably, *Hd_18862* is predominantly expressed in stems and leaves, mirroring the tissue distribution of geniposide and deacetyl asperulosidic acid methyl ester. This suggests it may function as an enzyme synthesizing both iridoids (**Figure 7H**). Previous research has identified O-acetyltransferase-like enzymes, such as Deacetylindoline O-acetyltransferase (Q9ZTK5.1) and Stemmadenine O-acetyltransferase (A0A2P1GIW7.1), in the biosynthesis of monoterpenoid indole alkaloids (MIA) downstream of secologanin (St-Pierre et al., 1999; Qu et al., 2018). However, the homology between OAT candidate genes and these genes is low, despite the clustering of some OAT candidate genes with them. This suggests that the biosynthesis of asperlosidic acid may involve additional complex regulatory mechanisms (**Figure 7F**).

The cytochrome P450 (CYP450) gene family plays a pivotal role in regulating plant chemical diversity (Mizutani and Sato, 2011). This family is extensively involved in the biosynthesis of various terpenes, with the CYP71 subfamily being particularly significant in mediating redox processes of monoterpenes and sesquiterpenes (Drew et al., 2013; Yuting et al., 2020; Xuan et al., 2024). In this study, we propose that the C-6 hydroxylation of the target iridoids (deacetyl asperulosidic acid methyl ester and deacetyl asperulosidic acid) is catalyzed by members of the CYP71 subfamily (**Figure 7A**). Phylogenetic analysis identified two CYP71 candidate genes, *Hd_18118* and *Hd_18119*, which clustered closely with CYP71D55 (A6YIH8.1) (**Figure 7G**). CYP71D55 is known to catalyze the hydroxylation of sesquiterpenes, specifically converting valencene to nootkatol (Park et al., 2022). In addition, in the study of sesquiterpene synthase in Solanaceae, CYP71D55 is functionally characterized as a terpene hydroxylase, which can catalyze the mono hydroxylation of valencene to nootkatol (Takahashi et al., 2007). Furthermore, functional characterization of CYP71D55 in Solanaceae sesquiterpene synthase studies has confirmed its role as a terpene hydroxylase, facilitating the mono-hydroxylation of valencene to nootkatol (Wenger et al., 2019). We hypothesize that this hydroxylation mechanism on unsaturated rings may similarly influence iridoids C-6 hydroxylation. Notably, *Hd_18118* and *Hd_18119* exhibit distinct expression patterns aligning with the production of Deacetyl asperlosidic acid and Deacetyl asperulosidic acid methyl ester, respectively. This suggests that in Hd, *Hd_18118* and *Hd_18119* may have functions similar to CYP71D55, catalyzing the production of Deacetyl asperlosidic acid and Deacetyl asperulosidic acid methyl ester (**Figure 7H**).

## Conclusions

In summary, we have presented crucial genomic data for Hd, a significant medicinal plant in traditional medicine, and reported a 482.30Mb T2T genome with 16 chromosomes. Hd underwent a whole-genome duplication event around 32 MYA. Our multiomic analysis elucidated the regulatory roles of pertinent coding genes in the biosynthetic pathway of iridoids metabolites. Additionally, we predicted one LAMT and two CYP71D55 genes as pivotal enzymes in the potential post-modification process of iridoids. This study contributes to a better understanding of the intricate iridoids biosynthesis pathway and enhances the economic value of Hd as a medicinal plant.

## Materials and methods

### Plant materials

The Hd samples analyzed in this study were sourced from a traditional Chinese medicine cultivation site in Xinzhou City, Hubei Province. These samples were confirmed to be genuine Hd specimens. High-quality DNA was extracted from fresh plant leaves using the QIAGEN Genomic testing kit (**Figure 2A**).

### Genome sequencing

We employed the HiFi Reads technology from the PacBio sequencing platform, known for its long read length and high accuracy, to enhance mutation detection, expedite assembly, and improve the continuity, accuracy, and completeness of genome assembly (Wenger et al., 2019). Initially, DNA quality was assessed using 0.75% agarose electrophoresis, Nanodrop, and Qubit methods. Subsequently, genomic DNA underwent cleavage with g-TUBE (Covaris, USA), followed by enrichment and purification of target DNA fragments using magnetic beads. DNA polymerase was utilized for repairing fragmented DNA and linking stem-loop sequencing adapters at both ends. Target fragments were then screened and purified using the gel cutter BluePippin (Sage Science, USA) to generate the library. The size of library fragments was assessed using the Agilent 2100 Bioanalyzer (Agilent Technologies, USA). Subsequently, samples were loaded onto the nanopores of the PacBio Sequel II series sequencer for real-time single-molecule sequencing to acquire raw sequencing data. Finally, the HQRF (High-Quality Region Finder) in the Smrtlink software was employed to identify the longest region of single enzyme activity and eliminate low-quality regions based on SNR. Read data exceeding 1000bp in length were selected for assembly.

### Chromosome assembly by Hi-C data

Sequencing the Hi-C library directly enables the acquisition of comprehensive chromatin interaction data essential for constructing chromosome-level genomic super scaffolds (Belton et al., 2012). Fresh leaves of Hd were dissected into 2 cm segments and fixed with 2% formaldehyde for genomic DNA extraction to build a Hi-C fragment library. Quality control of the Hi-C raw data involved filtering out low-quality sequences, bridging sequences, and sequences shorter than 30 bp with a quality score <20. Utilizing bowtie2 (v2.3.2) facilitated obtaining uniquely mapped paired-end reads, followed by the identification and retention of valid interacting read pairs from these uniquely mapped reads using HiC-Pro (v2.8.1), which also eliminated invalid read pairs. Subsequently, LACHESIS was employed for further aggregation, sorting, and orientation of scaffolds onto chromosomes, employing parameters including CLUSTER, MIN-RESITES=100, CLUSTER-MAX_LINK_DENSITY=2.5, CLUSTER NONFORMATIVE RATIO=1.4, and ORDER.

### Genome assembly and quality assessment

The genome assembly of third-generation sequencing data was conducted using Hifiasm software to achieve high-precision genomes. Decontamination and redundancy analyses were performed based on existing genomic background data to assess genome quality. Specifically, the Smrtlink software, developed independently from the PacBio platform, was utilized to identify the longest region of single enzyme activity through HQRF. Subsequently, low-quality regions of the genome were filtered using SNR. The CCS software was employed to convert subreads into HiFi reads for quality control, followed by filtering reads longer than 1000 bp for direct assembly. The quality of the assembled high-precision genome by Hifiasm was evaluated using BUSCO, CEGMA, and genomic quality assessment methods such as sequence consistency evaluation and GC deep analysis.

### Gene structure and functional annotation

Concatenated repeat sequences were identified using GMAA30 and TRF (Tandem Repeat Sequence Finder) with default parameters (Benson, 1999; Wang and Wang, 2016). Duplicate sequences were searched using MITE hunter software (Han and Wessler, 2010), LTR finder (Xu and Wang, 2007) and ltr harperst (Ellinghaus et al., 2008). RepeatMask was employed to detect repetitive sequences across the genome. Gene structures were defined by integrating *de novo* prediction, homologous annotation, and transcriptome prediction methods with EVM. Genes containing transposable elements were analyzed using TransposonPSI. Prediction of non-coding RNA sequences, including rRNA, snRNA, and miRNA, was conducted by comparing submissions to the Rfam database using cmscan in Inferna software. Functional annotation of protein-coding genes was performed using BLASTP and InterproScan software. Protein sequences were compared to the NR, KEGG, SwissProt, GO, and KOG databases to merge protein annotation information.

### Gene family and Phylogenomic analysis

To investigate the evolutionary patterns of Hd, we identified protein sequences of dicotyledonous plants, including Hd, using Orthofinder v2.5.4 software and constructed a phylogenetic tree based on single-copy genes. Subsequently, we aligned the sequences of each single-copy gene family using MAFFT v7.20535 (Katoh et al., 2009), followed by the conversion of aligned protein sequences into codon alignments using the PAL2NAL v14 program (Suyama et al., 2006). For model selection, IQ-TREE was employed, identifying the JTT+F+R4 model as optimal. A maximum likelihood (ML) phylogenetic tree was constructed with 1000 bootstrap replicates, achieving a bootstrap support rate of 100%. *Dioscorea alata* was designated as the outgroup, and divergence times were estimated using the MCMCTREE package in PAML v4.9i software.

### Investigation of whole-genome duplication

To identify whole-genome duplication (WGD) events in Hd, we initially employed diamond v2.0.337 (Buchfink et al., 2015) to align gene sequences across species and detect orthologous gene pairs. Subsequently, collinearity maps of genes were generated using WGDI software. Utilizing the blast method for protein homology alignment and MCScanX for homologous gene pair identification, we computed the synonymous substitution rate (Ks) and constructed Ks curves to analyze synonymous mutation rates within and between species. Integration of these analyses allows for inference of WGD events in the focal species.

### Metabolomic analysis

Samples of Hd from various plant parts (roots, stems, leaves, flowers, fruits) were selected for metabolic analysis (n=6). Metabolites were detected using a comprehensive metabolomics approach based on UPLC-MS/MS on an Agilent Ultra High Performance Liquid Chromatography System (ExionLC™ AD) with a self-constructed database. The chromatographic column employed was an Agilent SB-C18 (1.8 µm * 2.1 mm * 100 mm), with a flow rate of 0.35 mL/min. The mobile phase consisted of ultrapure water and acetonitrile, both containing 0.1% formic acid, with an initial gradient elution ratio of 5% ultrapure water. Plant samples were freeze-dried, ground into powder, and 50 mg of the sample was weighed. Subsequently, 1200 μl of pre-cooled 70% methanol water internal standard extraction solution was added, followed by vortexing and centrifugation (12000 rpm, 3 min). The supernatant was aspirated, and a 0.22 μl sample solution was filtered through a microporous filter membrane for analysis.

The mass spectrometry parameters were set as follows: Electrospray Ionization (ESI) temperature was maintained at 500°C; Ion spray voltage (IS) was set at 5500 V in positive ion mode and -4500 V in negative ion mode; The ion source gases I (GSI), II (GSII), and curtain gas (CUR) were adjusted to 50, 60, and 25 psi, respectively. Collision-induced ionization settings were optimized to high levels. The QQQ scan operated in Multiple Reaction Monitoring (MRM) mode with medium collision gas (nitrogen). Declustering potential (DP) and collision energy (CE) were fine-tuned for each MRM ion pair. A specific set of MRM ion pairs corresponding to eluting metabolites was monitored in each period. Data analysis was performed using Analyst 1.6.3 software, and the relative content of each component was determined using peak area normalization. The samples’ metabolites were analyzed qualitatively and quantitatively using mass spectrometry based on a local metabolic database. Detection employed multi-response monitoring mode (MRM) to identify characteristic ions for each substance through a quadrupole mass analyzer. Signal strength (CPS) of these ions was recorded. Subsequently, the mass spectrometry data was processed using MultiQuant software for peak integration and correction. The peak area of each chromatographic peak reflected the relative content of the corresponding metabolite. Integrated data of all peak areas were saved. To compare metabolite content differences among samples, chromatographic peaks were adjusted based on metabolite retention time and peak type to ensure qualitative and quantitative accuracy.

### Transcriptomic analysis

Total RNA was extracted from roots, stems, leaves, flowers, and fruits of Hd using the HiPure Plant RNA Kit (Magen, Guangzhou, China) for RNA sequencing. Raw sequencing data were processed using Fastp software (https://github.com/OpenGene/fastp) to filter low-quality reads. The quality of the cleaned data was assessed using FastQC (http://www.bioinformatics.babraham.ac.uk/projects/fastqc). Clean reads were then aligned to the reference genome using HISAT2 software. Gene expression levels were quantified as FPKM (fragments per kilobase of transcript per million mapped reads). Differential expression analysis was conducted using DESeq2 software, with thresholds set at |log2(FoldChange)| ≥ 2 and adjusted p-value (*P*adj) ≤ 0.05.

Pairwise comparisons were performed across different tissue types, and differentially expressed genes (DEGs) were subjected to Gene Ontology (GO) and Kyoto Encyclopedia of Genes and Genomes (KEGG) enrichment analyses, with significance determined by corrected p-values (*P*adj < 0.05). Data visualization was performed using the ChiPlot bioinformatics platform (https://www.chiplot.online/). In GO and KEGG analyses, “count” refers to the number of genes annotated in the respective enriched terms.

### Identification of post-modified candidate genes

To identify the candidate genes involved in the post-biosynthetic modification processes (e.g., methylation, acetylation, hydroxylation) of iridoids, we constructed clustering heatmaps for the candidate genes of three key enzymes (LAMT, OAT, CYP71) based on their expression patterns across five tissues. A phylogenetic tree of the candidate genes was generated using MEGA11 software. The protein sequences of the candidate genes were aligned with those from other plant species (*Catharanthus roseus, Hyoscyamus muticus, Hamelia patens, Mitragyna* sp*eciosa*) using Clustal with default parameters. Subsequently, a phylogenetic tree was constructed using the neighbor-joining method with 1,000 bootstrap replicates. Clustering with known functional genes was performed to infer evolutionary relationships and functional similarities. Additionally, BLASTP analysis (https://blast.ncbi.nlm.nih.gov/Blast.cgi) was conducted to evaluate the sequence similarity between the candidate genes and reference enzymes. The term “Per.ident” refers to the percentage of sequence identity in alignments, which serves as a metric for assessing alignment consistency. We propose that a “Per.ident” value exceeding 60% provides evidence, to some extent, that the candidate gene may possess a function similar to that of the reference protein.

### Analysis of qRT-PCR

RNA quality assessment was conducted prior to extracting 1μg of total RNA from each sample. The extracted total RNA was then reverse-transcribed into cDNA using the PrimeScript™ 1st strand cDNA Synthesis Kit.Add the following components to a tube placed in an ice bath: 1 μg of total RNA, 1 μl of Oligo(dT) primer (50 μM), and 1 μl of dNTP Mix (10 mmol/L) were combined with RNase-free dH2O to a total volume of 10 μl. The mixture was then incubated at 65 °C for 5 minutes followed by rapid cooling on ice. Subsequently, add the following components to the same tube in the ice bath: 10 μl of Template RNA Primer Mixture (obtained from the previous step), 4 μl of 5×Reaction Buffer, 0.5 μl of RNase Inhibitor (40 U/μl), 1 μl of MMLV RT (200 U/μl), and 20 μl of RNase-free dH2O. The reaction mixture was then incubated at 42 °C for 30-60 minutes and terminated by heating at 95 °C for 5 minutes. For the PCR reaction setup, prepare a mixture consisting of 10 μl of 2× SYBR real-time PCR premixture, 0.4 μl of 10 μM PCR specific primer F, 0.4 μl of 10 μM PCR specific primer R, 1 μl of cDNA, and 20 μl of RNase-free dH2O. Perform an initial denaturation at 95 °C for 5 minutes, followed by 30 cycles of denaturation at 95 °C for 15 seconds, annealing at 60 °C for 30 seconds. The relative content was determined using the 2^-ΔΔCt^ analysis method with the formula: Ratio = 2− [CT (test)− CT (calibrator)] (test/calibrator).

## Data Availability

The datasets presented in this study can be found in online repositories. The names of the repository/repositories and accession number(s) can be found below: https://www.ncbi.nlm.nih.gov/, PRJCA036707.
