# Supplementary material for: The Hedyotis diffusa chromosome-level genome and multi-omics analysis provide new insights into the iridoids biosynthetic pathway
Source: Front Plant Sci. 2025 Jun 19;16:1607226. doi: 10.3389/fpls.2025.1607226 (PMC12222090; doi:10.3389/fpls.2025.1607226)
Supplement: Supplementary file 1 [file DataSheet1.docx]

**The *Hedyotis diffusa* chromosome-level genome and multi-omics analysis provide new insights into the iridoids biosynthetic pathway**

*Pengyu Chen et al.*


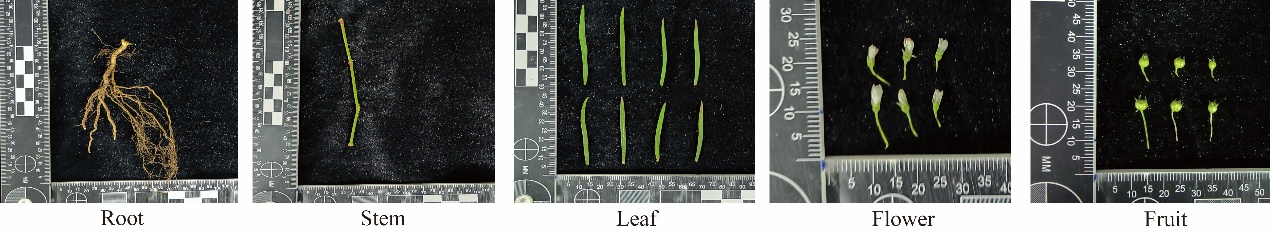


**Figure S1** Photos of samples from various tissues of *Hedyotis diffusa*, including Roots, Stems, Leaves, Flowers, and Fruits.


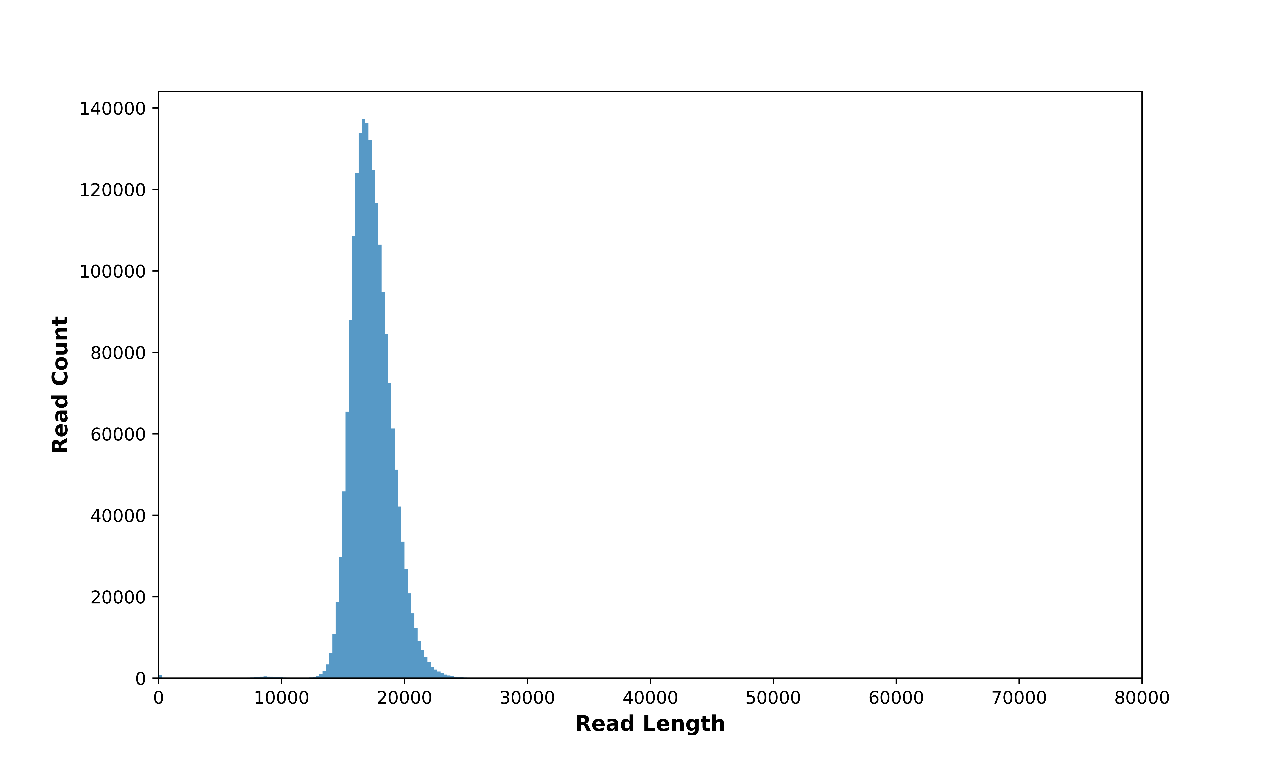


**Figure S2** Histogram of read length distribution for sequencing data


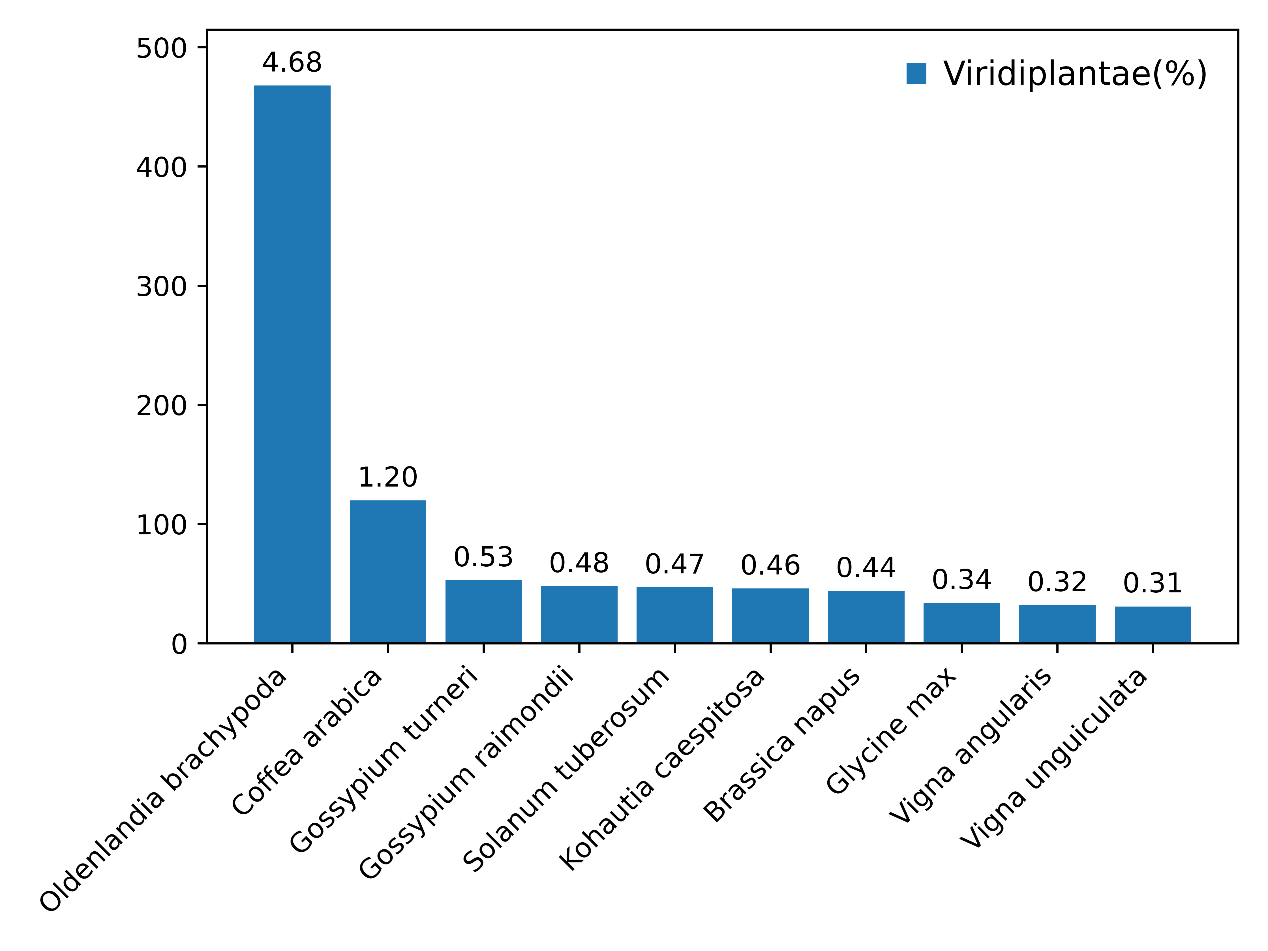


**Figure S3** Distribution map of main species（TOP 10）


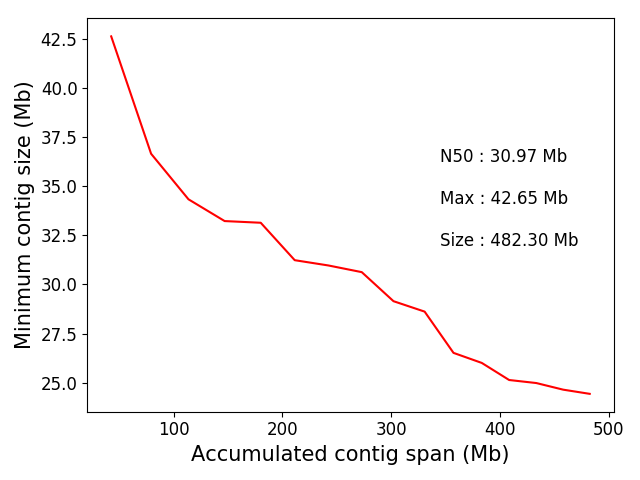


**Figure S4** Contig sequence length cumulative graph


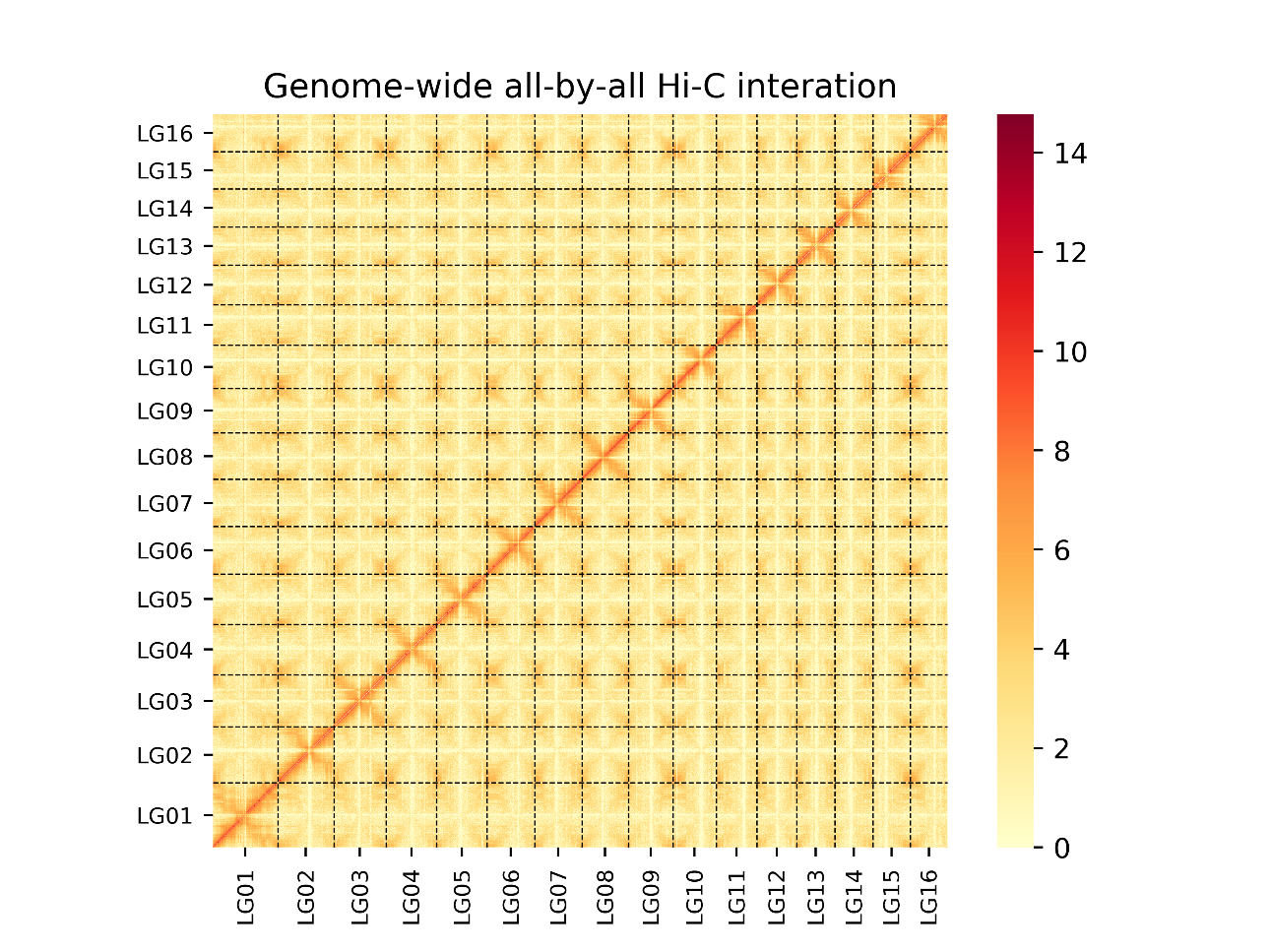


**Figure S5** Hi-C interaction heatmap of chromosomes


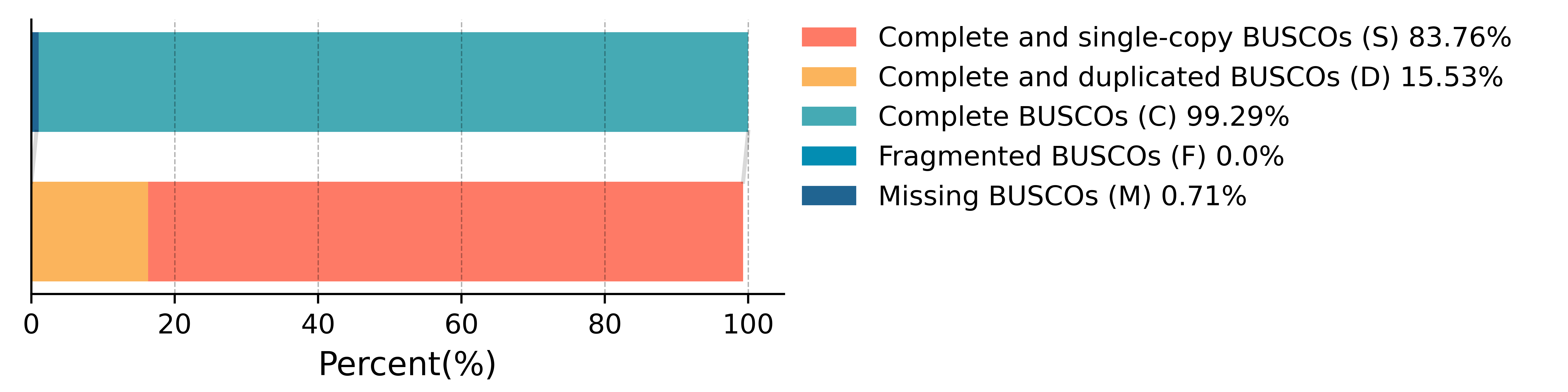


**Figure S6** BUSCO analysis


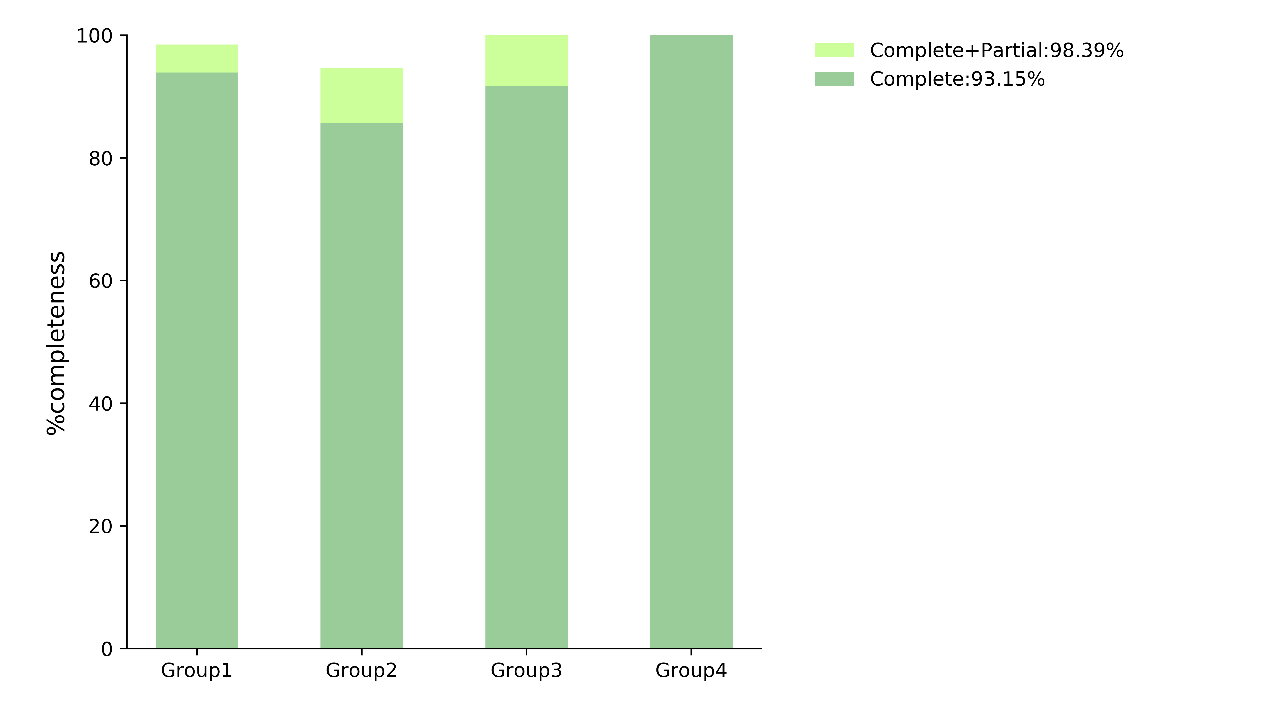


**Figure S7** CEGMA assessment


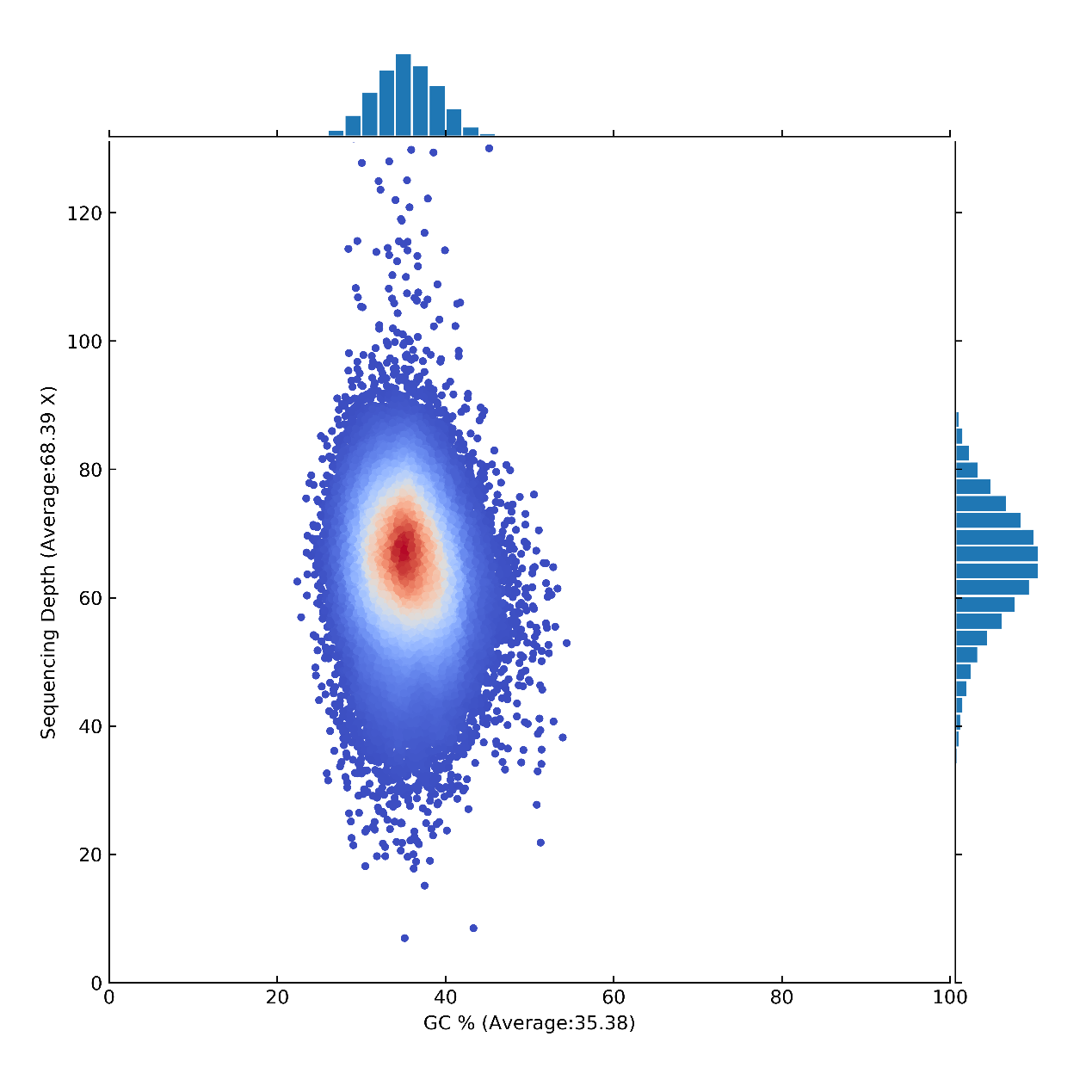


**Figure S8** GC Depth Distribution


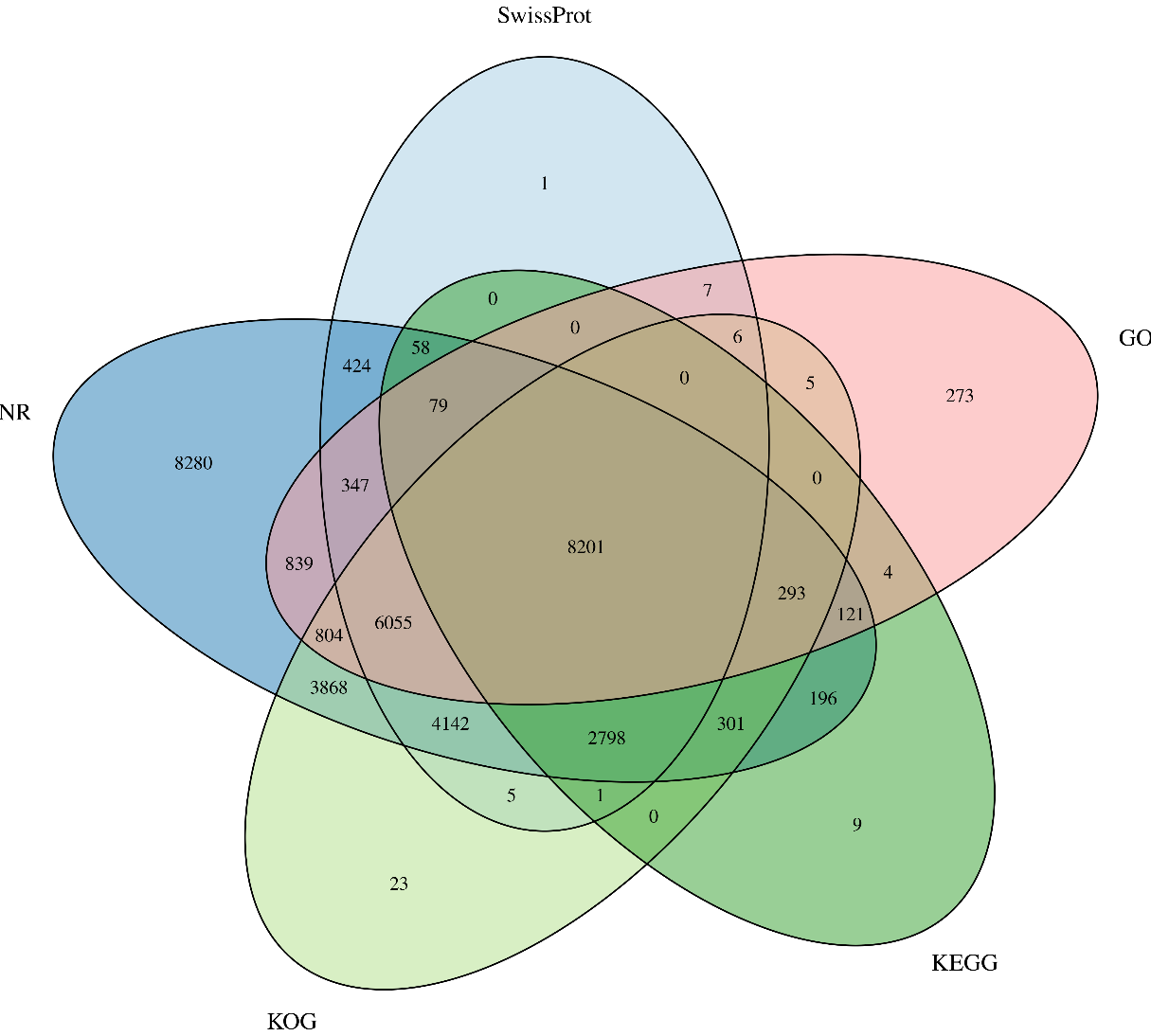


**Figure S9** Venn diagram of annotation results for each database


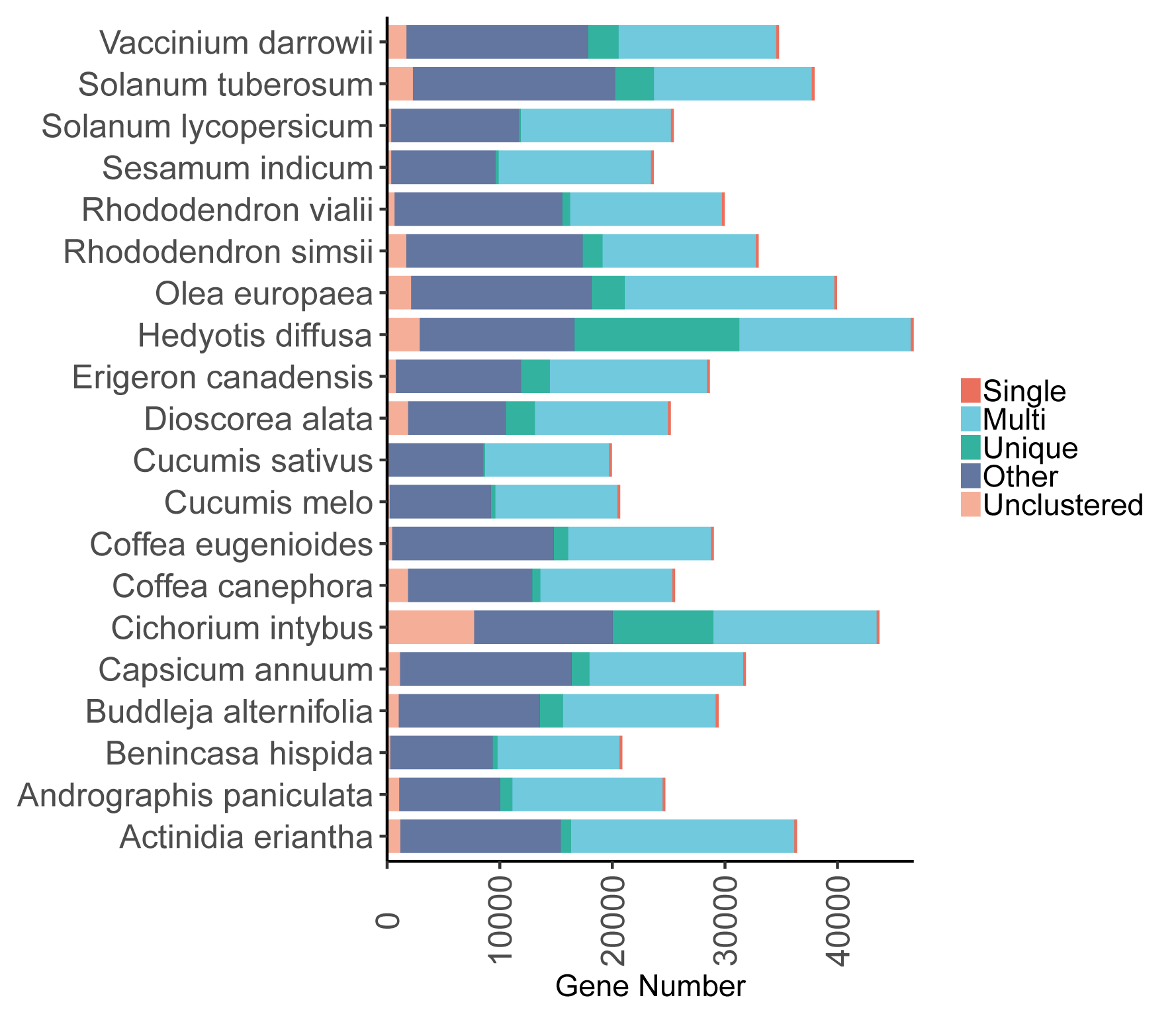


**Figure S10** Gene distribution maps of different types of all species


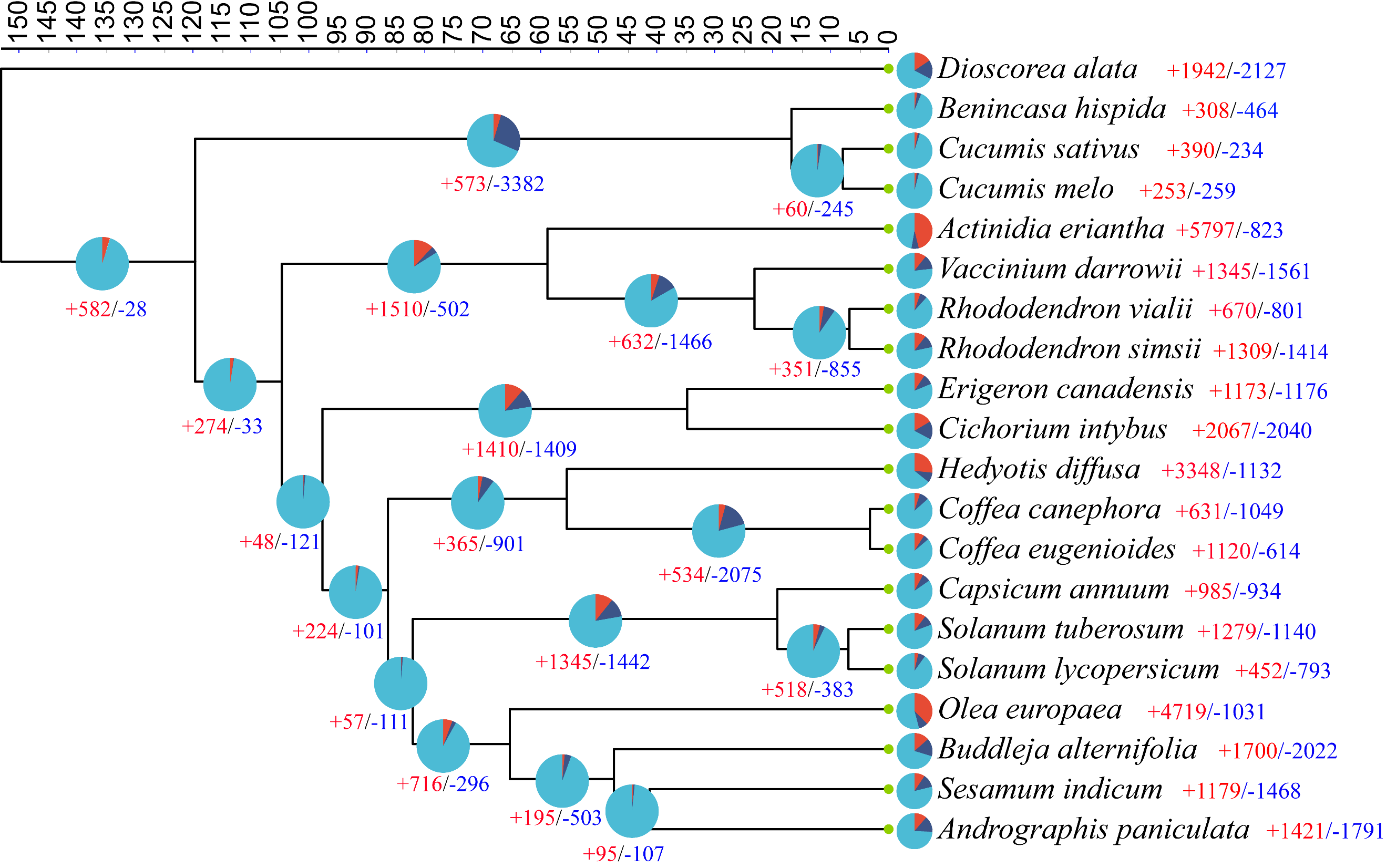


**Figure S11** The contraction and expansion of gene families of various species based on evolutionary tree display in *Hedyotis diffusa.* In the circular scale map, the light blue fan represents the conserved gene family, the red fan represents the expanded gene family, and the dark blue fan represents the contracted gene family


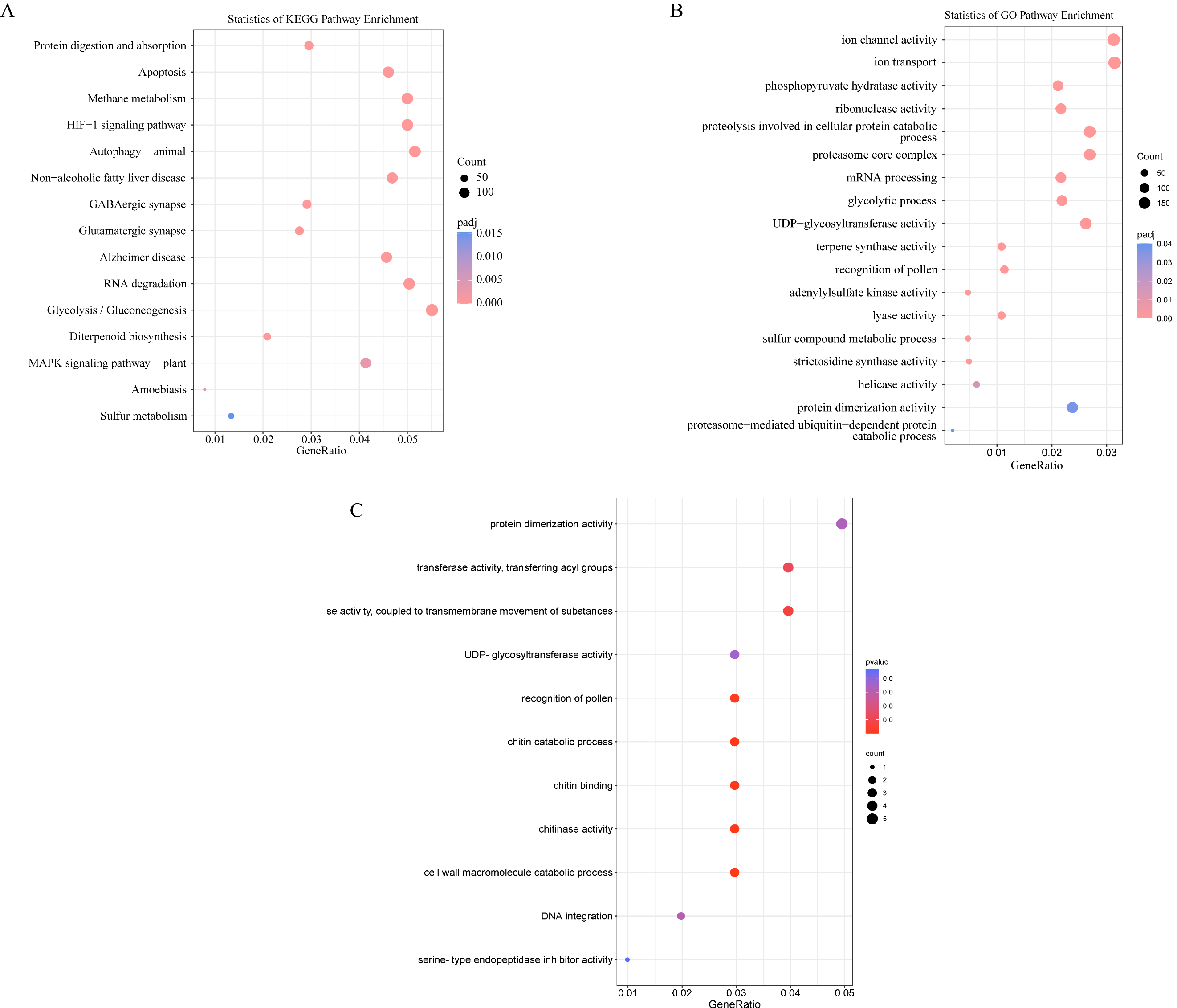


**Figure S12** Gene functional enrichment analysis. A: KEGG enrichment analysis of unique genes in *Hedyotis diffusa*; B: GO enrichment analysis of unique genes in *Hedyotis diffusa* ; C:GO analysis of the expansion gene family in *Hedyotis diffusa*


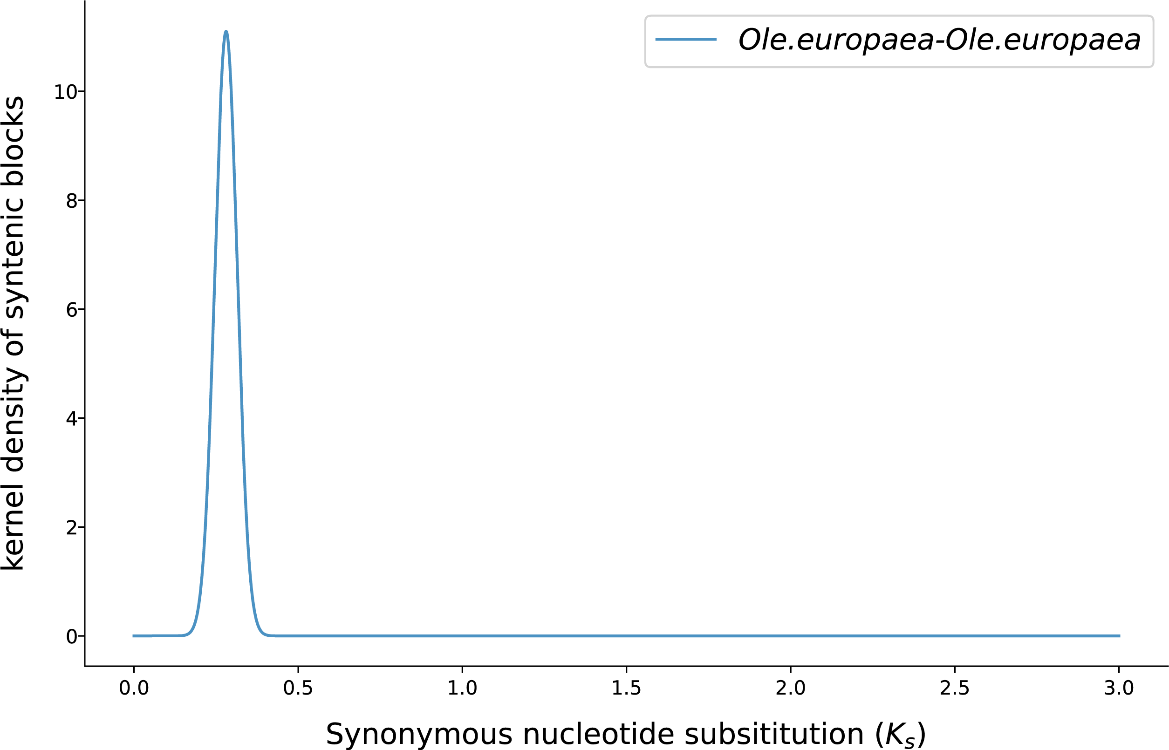


**Figure S13** the synonymous nucleotide subsititution of Olea europaea

**
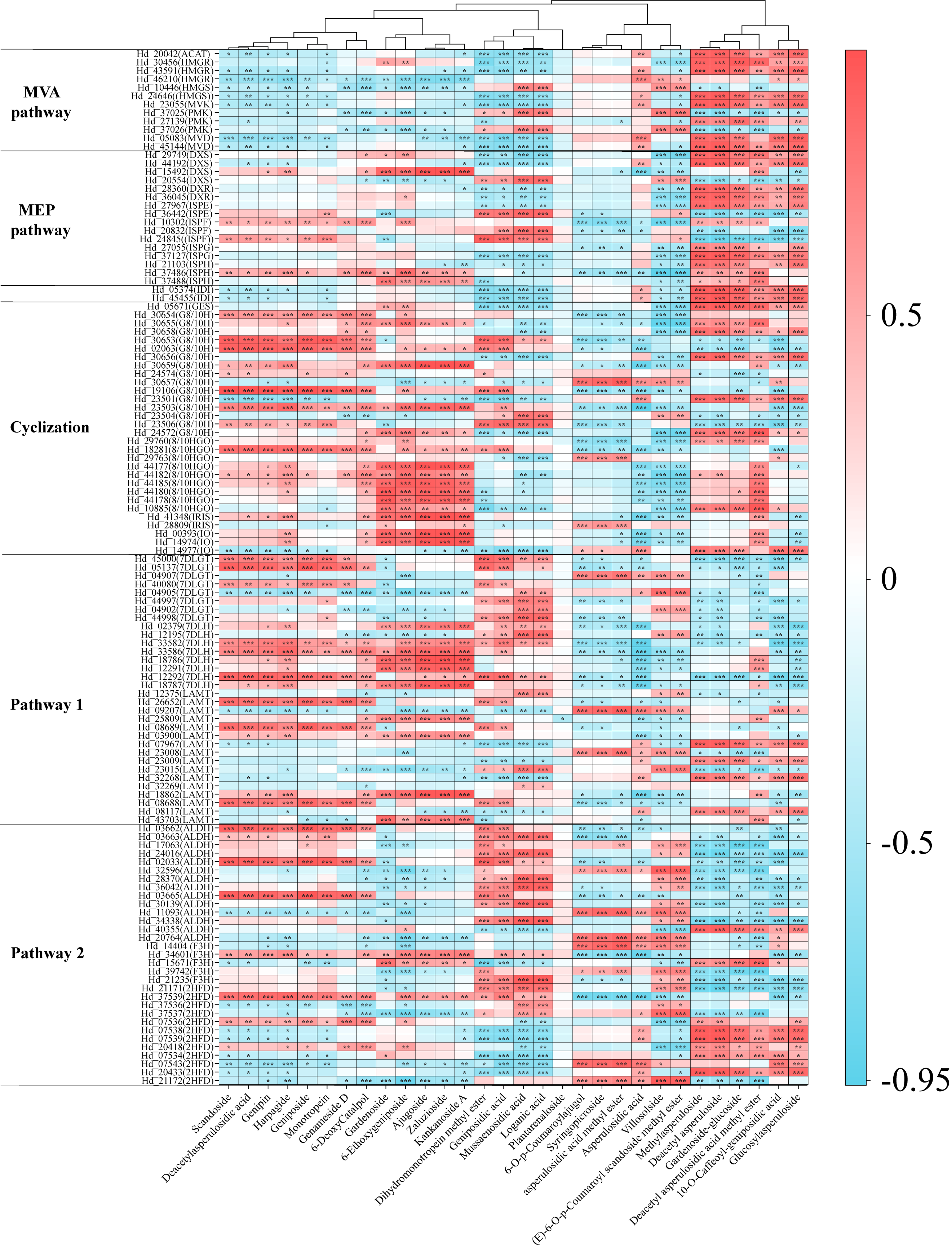
**

**Figure S14** Correlation analysis heat map of 30 iridoid metabolites with iridoid biosynthesis pathway genes.

**Table S1** Quality control statistics of third-generation sequencing data

| Type | Raw Reads | Filtered Reads |
| --- | --- | --- |
| Bases(bp) | 33,799,192,171 | 33,799,059,475 |
| Reads number | 1,952,045 | 1,951,089 |
| Reads mean length(bp) | 17,314.76 | 17,323.18 |
| Reads max length(bp) | 37,401 | 37,401 |
| Reads N50 (bp) | 17,327 | 17,327 |
| Reads >10kb ratio(%) | 99.68 | 99.68 |
| Reads >20kb ratio(%) | 5.79 | 5.79 |
| Reads >40kb ratio(%) | 0 | 0 |

**Table S2** Species distribution statistics（TOP 10）

| Species | Reads Number | Taxonomy |
| --- | --- | --- |
| Unmap | 7615 | Unknow |
| Oldenlandia brachypoda | 468 | Viridiplantae |
| Coffea arabica | 120 | Viridiplantae |
| Gossypium turneri | 53 | Viridiplantae |
| Gossypium raimondii | 48 | Viridiplantae |
| Solanum tuberosum | 47 | Viridiplantae |
| Kohautia caespitosa | 46 | Viridiplantae |
| Brassica napus | 44 | Viridiplantae |
| Glycine max | 34 | Viridiplantae |
| Vigna angularis | 32 | Viridiplantae |
| Vigna unguiculata | 31 | Viridiplantae |

Annotation：Unmap indicates that reads from existing sequences in the NT library have not been matched

**Table S3** Species distribution statistics（TOP 10）

| StatType | Contig Length | Contig Number |
| --- | --- | --- |
| N50 | 30967793 | 7 |
| N60 | 29147419 | 9 |
| N70 | 26512958 | 11 |
| N80 | 25129718 | 13 |
| N90 | 24638054 | 15 |
| Longest | 42645787 | 1 |
| Total | 482301528 | 16 |
| Length>=1kb | 482301528 | 16 |
| Length>=2kb | 482301528 | 16 |
| Length>=5kb | 482301528 | 16 |

**Table S4** 16 chromosome length statistics based on Contig clustering

| Chr | Length(bp) | Contig Num |
| --- | --- | --- |
| LG01 | 42645787 | 1 |
| LG02 | 36661983 | 1 |
| LG03 | 34340531 | 1 |
| LG04 | 33231257 | 1 |
| LG05 | 33145331 | 1 |
| LG06 | 31237536 | 1 |
| LG07 | 30967793 | 1 |
| LG08 | 30627819 | 1 |
| LG09 | 29147419 | 1 |
| LG10 | 28615796 | 1 |
| LG11 | 26512958 | 1 |
| LG12 | 26000403 | 1 |
| LG13 | 25129718 | 1 |
| LG14 | 24974879 | 1 |
| LG15 | 24638054 | 1 |
| LG16 | 24424264 | 1 |
| Total | 482301528 | 16 |

**Table S5** BUSCO Forecast Statistics Table

| Type | Number | Percent(%) |
| --- | --- | --- |
| Complete BUSCOs (C) | 422 | 99.29 |
| Complete and single-copy BUSCOs (S) | 356 | 83.76 |
| Complete and duplicated BUSCOs (D) | 66 | 15.53 |
| Fragmented BUSCOs (F) | 0 | 0 |
| Missing BUSCOs (M) | 3 | 0.71 |
| Viridiplantae | 425 | 100 |

**Table S6** BUSCO Forecast Statistics Table

| Depth(X) | Heteo SNP | Hetero Indel | Homo SNP | Homo Indel | Accuracy of genome(%) |
| --- | --- | --- | --- | --- | --- |
| depth>=1x | 239834 | 18,132 | 1,040,740 | 137,921 | 99.755617 |
| depth>=5x | 238,650 | 16,666 | 1,013,984 | 128,572 | 99.763103 |
| depth>=10x | 235,107 | 13,429 | 989,525 | 121,825 | 99.769574 |

**Table S7** Genome coverage statistics

| Depth（X） | Base number | Coverage ratio(%) |
| --- | --- | --- |
| 1 | 482,298,653 | 100.00 |
| 5 | 482,290,455 | 100.00 |
| 10 | 482,265,876 | 99.99 |
| 20 | 482,105,449 | 99.96 |
| Coverage depth(X) | 68.39 | |

**Table S8** Genome telomere location and length distribution

| Seq ID | Seq length | Start | End | Telomere length | Start | End | Telomere length |
| --- | --- | --- | --- | --- | --- | --- | --- |
| tig01 | 42645787 | 1 | 4642 | 4642 | 42640518 | 42645787 | 5270 |
| tig02 | 36661983 | 1 | 3629 | 3629 | 36656911 | 36661983 | 5073 |
| tig03 | 34340531 | 1 | 4418 | 4418 | 34336501 | 34340531 | 4031 |
| tig04 | 33231257 | 1 | 3958 | 3958 | 33226684 | 33231257 | 4574 |
| tig05 | 33145331 | 1 | 3366 | 3366 | 33141070 | 33145331 | 4262 |
| tig06 | 31237536 | 1 | 2875 | 2875 | 31235172 | 31237536 | 2365 |
| tig07 | 30967793 | - | - | - | 30963161 | 30967793 | 4633 |
| tig08 | 30627819 | 1 | 4623 | 4623 | 30624076 | 30627819 | 3744 |
| tig09 | 29147419 | 1 | 372 | 372 | 29143436 | 29147419 | 3984 |
| tig10 | 28615796 | 1 | 3096 | 3096 | 28611972 | 28615796 | 3825 |
| tig11 | 26512958 | 1 | 5907 | 5907 | 26508872 | 26511813 | 2942 |
| tig12 | 26000403 | 1 | 7082 | 7082 | 25996733 | 26000403 | 3671 |
| tig13 | 25129718 | 1 | 3740 | 3740 | 25125513 | 25129718 | 4206 |
| tig14 | 24974879 | 1 | 2297 | 2297 | 24971916 | 24974342 | 2427 |
| tig15 | 24638054 | - | - | - | 24634445 | 24638054 | 3610 |
| tig16 | 24424264 | 1 | 456 | 456 | 24419604 | 24424264 | 4661 |

**Table S9** Gene Function Annotation Statistics

| Type | Number | Percent (%) |
| --- | --- | --- |
| Pfam | 28,706 | 61.39 |
| KEGG | 12,061 | 25.79 |
| SwissProt | 22,124 | 47.31 |
| KOG | 26,502 | 56.68 |
| GO | 17,034 | 36.43 |
| TIGRFAMs | 3,233 | 6.91 |
| NR | 36,806 | 78.71 |
| At least one databases | 37,644 | 80.51 |
| Overall | 46,759 | 100.00 |

**Table S10** BUSCO Forecast Statistics

| Type | Number | Percent(%) |
| --- | --- | --- |
| Complete BUSCOs (C) | 416 | 97.88 |
| Complete and single-copy BUSCOs (S) | 368 | 86.59 |
| Complete and duplicated BUSCOs (D) | 48 | 11.29 |
| Fragmented BUSCOs (F) | 1 | 0.24 |
| Missing BUSCOs (M) | 8 | 1.88 |
| Viridiplantae | 425 | 100 |

**Table S11** Non-coding RNA annotation statistics

| Class | Type | Number | Average length(bp) | Total length(bp) | Percentage(%) |
| --- | --- | --- | --- | --- | --- |
| tRNA | - | 580 | 75.18 | 43,607 | 0.009 |
| rRNA | - | 412 | 244.34 | 100,668 | 0.0209 |
|  | 5S | 374 | 115.43 | 43,172 | 0.009 |
|  | 5.8S | 7 | 147.43 | 1,032 | 0.0002 |
|  | 18S | 13 | 1,468.69 | 19,093 | 0.004 |
|  | 28S | 10 | 1,954.60 | 19,546 | 0.0041 |
| Small ncRNA | - | 41 | 139.73 | 5,729 | 0.0012 |
|  | miRNA | 0 | 0 | 0 | 0 |
|  | siRNA | 0 | 0 | 0 | 0 |
| Medium ncRNA | - | 210 | 96.8 | 20,327 | 0.0042 |
|  | snoRNA | 0 | 0 | 0 | 0 |
|  | snRNA | 210 | 96.8 | 20,327 | 0.0042 |
| Other | - | 222 | 135.4 | 30,059 | 0.0062 |
| Total | - | 1,465 | 136.78 | 200,390 | 0.0415 |

**Table S12** Statistical results of TE repeat sequences

| Class | Order | Super family | Number of elements | Length of sequence (bp) | Percentage of sequence (%) |
| --- | --- | --- | --- | --- | --- |
| Class I |  |  | 66,443 | 67,975,192 | 14.09 |
|  | LTR |  | 57,732 | 66,410,398 | 13.77 |
|  |  | LTR/Gypsy | 36,306 | 38,894,959 | 8.06 |
|  |  | LTR/Copia | 17,688 | 24,789,386 | 5.14 |
|  |  | LTR/unknown | 3,492 | 2,656,397 | 0.55 |
|  |  | LTR/forward | 134 | 40,885 | 0.01 |
|  |  | LTR/Caulimovirus | 82 | 19,566 | 0 |
|  |  | LTR/reverse | 11 | 6,037 | 0 |
|  |  | LTR/Cassandra | 1 | 59 | 0 |
|  | SINE |  | 7,272 | 1,253,763 | 0.26 |
|  |  | SINE/tRNA-RTE | 17 | 1,847 | 0 |
|  | LINE |  | 1,439 | 311,031 | 0.06 |
|  |  | LINE/L1 | 1,191 | 216,358 | 0.04 |
|  |  | LINE/RTE-BovB | 166 | 82,495 | 0.02 |
|  |  | LINE/reverse | 58 | 8,304 | 0 |
|  |  | LINE/forward | 21 | 3,726 | 0 |
|  |  | LINE/RTE-X | 2 | 110 | 0 |
|  |  | LINE/Dualen | 1 | 38 | 0 |
| Class II |  |  | 250,304 | 116,143,509 | 24.08 |
|  | DNA |  | 2,762 | 963,102 | 0.2 |
|  |  | DNA/PIF-Harbinger | 333 | 136,472 | 0.03 |
|  |  | DNA/MULE-MuDR | 382 | 60,899 | 0.01 |
|  |  | DNA/hAT-Tip100 | 468 | 246,646 | 0.05 |
|  |  | DNA/CMC-EnSpm | 430 | 103,061 | 0.02 |
|  |  | DNA/hAT-Tag1 | 177 | 75,336 | 0.02 |
|  |  | DNA/hAT-Ac | 543 | 189,783 | 0.04 |
|  |  | DNA/TcMar-Stowaway | 45 | 23,682 | 0 |
|  |  | DNA/TcMar-Pogo | 70 | 9,219 | 0 |
|  |  | DNA/forward | 230 | 104,838 | 0.02 |
|  |  | DNA/reverse | 35 | 7,077 | 0 |
|  |  | DNA/hAT | 1 | 61 | 0 |
|  |  | DNA/hAT-Charlie | 19 | 1,617 | 0 |
|  |  | DNA/Novosib | 1 | 72 | 0 |
|  |  | DNA/P | 1 | 50 | 0 |
|  |  | DNA/Dada | 1 | 62 | 0 |
|  | MITE |  | 52,283 | 11,559,792 | 2.4 |
|  | RC |  | 189,879 | 81,120,177 | 16.82 |
|  |  | RC/Helitron | 189,879 | 81,120,177 | 16.82 |
|  | TIR |  | 5,380 | 22,500,438 | 4.67 |
| Tandem Repeats |  |  | 163,681 | 12,582,225 | 2.61 |
|  | TRF |  | 102,841 | 11,442,332 | 2.37 |
|  | SSR |  | 60,840 | 1,139,893 | 0.24 |
| Other Repeat |  |  | 166 | 37,753 | 0.01 |
|  | Unclassified |  | 100 | 28,955 | 0.01 |
|  | Simple repeats |  | 40 | 3,588 | 0 |
|  | Satellites |  | 13 | 947 | 0 |
|  | reverse |  | 5 | 3,452 | 0 |
|  |  | reverse/reverse | 5 | 3,452 | 0 |
|  | Retroposon |  | 3 | 239 | 0 |
|  | unclear |  | 4 | 472 | 0 |
|  |  | unclear/forward | 4 | 472 | 0 |
|  | ARTEFACT |  | 1 | 100 | 0 |
| Total Repeats |  |  | 480,594 | 196,738,679 | 40.79 |

**Table S13** Gene family clustering among 20 plant species.

| **Sample** | **Genes**  **Number** | **Genes**  **Number In**  **Families** | **Uncluster**  **ed Genes** | **Family**  **Number** | **Unique**  **Families**  **Number** | **Average Genes**  **Number Per Family** |
| --- | --- | --- | --- | --- | --- | --- |
| Actinidia eriantha | 36,406 | 35,245 | 1,161 | 14,037 | 925 | 2.51 |
| Andrographis paniculata | 24,707 | 23,640 | 1,067 | 13,014 | 1,072 | 1.82 |
| Benincasa hispida | 20,894 | 20,621 | 273 | 13,262 | 428 | 1.55 |
| Buddleja alternifolia | 29,434 | 28,408 | 1,026 | 13,797 | 2,032 | 2.06 |
| Capsicum annuum | 31,867 | 30,728 | 1,139 | 14,644 | 1,568 | 2.1 |
| Cichorium intybus | 43,721 | 35,997 | 7,724 | 15,016 | 8,934 | 2.4 |
| Coffea canephora | 25,574 | 23,724 | 1,850 | 14,436 | 712 | 1.64 |
| Coffea eugenioides | 29,016 | 28,578 | 438 | 14,295 | 1,264 | 2 |
| Cucumis melo | 20,695 | 20,474 | 221 | 13,438 | 389 | 1.52 |
| Cucumis sativus | 19,962 | 19,823 | 139 | 13,339 | 167 | 1.49 |
| Dioscorea alata | 25,189 | 23,326 | 1,863 | 13,098 | 2,572 | 1.78 |
| Erigeron canadensis | 28,657 | 27,886 | 771 | 13,485 | 2,579 | 2.07 |
| Hedyotis diffusa | 46,759 | 43,869 | 2,890 | 14,094 | 14,641 | 3.11 |
| Olea europaea | 39,960 | 37,845 | 2,115 | 14,384 | 2,937 | 2.63 |
| Rhododendron simsii | 32,999 | 31,306 | 1,693 | 14,877 | 1,761 | 2.1 |
| Rhododendron vialii | 29,988 | 29,335 | 653 | 14,638 | 699 | 2 |
| Sesamum indicum | 23,676 | 23,324 | 352 | 13,201 | 273 | 1.77 |
| Solanum lycopersicum | 25,454 | 25,102 | 352 | 14,080 | 164 | 1.78 |
| Solanum tuberosum | 37,965 | 35,679 | 2,286 | 15,078 | 3,465 | 2.37 |
| Vaccinium darrowii | 34,804 | 33,097 | 1,707 | 14,915 | 2,698 | 2.22 |

**Table S13** Metabolomic data of iridoids in *Hedyotis diffusa*

| **Index** | **Compounds** | **Formula** | **Class II** | **Root** | **Stem** | **Leaf** | **Flower** | **Fruit** |
| --- | --- | --- | --- | --- | --- | --- | --- | --- |
| Sasn002370 | Deacetyl asperulosidic acid methyl ester | C17H24O11 | Monoterpenoids | 5.82E+06 | 6.59E+06 | 1.32E+06 | 6.50E+05 | 1.59E+05 |
| Lmdp002381 | Genipin | C11H14O5 | Monoterpenoids | 8.04E+05 | 2.32E+06 | 4.75E+06 | 1.34E+06 | 8.34E+05 |
| mws1574 | Geniposidic acid | C16H22O10 | Monoterpenoids | 2.78E+04 | 8.03E+05 | 1.86E+06 | 1.46E+06 | 4.39E+05 |
| Lhjp111614 | Methylasperuloside | C19H24O11 | Monoterpenoids | 8.41E+05 | 3.40E+05 | 1.87E+05 | 1.14E+05 | 2.08E+05 |
| Sasn003030 | asperulosidic acid methyl ester | C19H26O12 | Monoterpenoids | 9.91E+04 | 3.59E+04 | 1.44E+05 | 3.29E+04 | 2.93E+05 |
| Sasn003265 | Deacetyl asperuloside | C16H20O10 | Monoterpenoids | 1.04E+08 | 4.47E+07 | 2.30E+07 | 8.69E+06 | 2.69E+07 |
| pmn001585 | Monotropein | C16H22O11 | Monoterpenoids | 3.75E+06 | 6.85E+06 | 3.11E+07 | 1.08E+07 | 4.55E+06 |
| Zbyn002745 | Loganic acid | C16H24O10 | Monoterpenoids | 7.30E+05 | 2.63E+06 | 3.86E+06 | 5.96E+06 | 1.78E+06 |
| Cmzn002437 | Scandoside | C16H22O11 | Monoterpenoids | 6.96E+05 | 1.79E+06 | 4.74E+06 | 1.09E+06 | 9.04E+05 |
| mws1565 | Geniposide | C17H24O10 | Monoterpenoids | 1.45E+05 | 8.44E+05 | 6.44E+06 | 3.42E+05 | 2.39E+05 |
| Sasn003392 | (E)-6-O-p-Coumaroyl scandoside methyl ester | C26H30O13 | Monoterpenoids | 4.53E+05 | 4.73E+05 | 6.30E+05 | 2.28E+06 | 2.52E+06 |
| Lhjp111633 | Glucosylasperuloside | C24H32O16 | Monoterpenoids | 2.82E+06 | 4.07E+05 | 8.69E+05 | 5.48E+05 | 1.36E+06 |
| Lmdn005113 | Syringopicroside | C24H30O11 | Monoterpenoids | 9.94E+05 | 8.67E+05 | 3.22E+05 | 6.79E+05 | 2.02E+06 |
| MWSmce434 | Gardenoside | C17H24O11 | Monoterpenoids | 1.04E+05 | 3.50E+05 | 2.89E+05 | 1.46E+05 | 1.13E+05 |
| pmn001586 | Deacetylasperulosidic acid | C16H22O11 | Monoterpenoids | 1.53E+07 | 4.10E+07 | 9.02E+07 | 2.58E+07 | 2.15E+07 |
| pmn001587 | Asperulosidic acid | C18H24O12 | Monoterpenoids | 5.00E+07 | 1.60E+07 | 1.86E+07 | 3.59E+07 | 5.30E+07 |
| Sasn002290 | 6-Ethoxygeniposide | C19H28O11 | Monoterpenoids | 8.79E+06 | 1.80E+07 | 1.28E+07 | 2.64E+06 | 1.27E+06 |
| Yajn003587 | Zaluzioside | C17H24O11 | Monoterpenoids | 8.87E+05 | 1.34E+07 | 5.45E+06 | 7.65E+05 | 7.29E+05 |
| Wmhp000012 | Ajugoside | C17H26O10 | Monoterpenoids | 1.18E+06 | 8.78E+06 | 3.81E+06 | 6.32E+05 | 8.49E+05 |
| Cmrn001591 | Mussaenosidic acid | C16H24O10 | Monoterpenoids | 4.44E+05 | 1.58E+06 | 2.23E+06 | 3.61E+06 | 1.11E+06 |
| Cmrn002988 | Kankanoside A | C16H26O8 | Monoterpenoids | 3.83E+05 | 4.50E+06 | 2.07E+06 | 1.10E+06 | 6.41E+05 |
| Wmbn000547 | Harpagide | C15H24O10 | Monoterpenoids | 3.34E+05 | 1.53E+06 | 2.47E+06 | 4.58E+05 | 2.70E+05 |
| Cmzp002600 | Dihydromonotropein methyl ester | C17H26O11 | Monoterpenoids | 1.70E+05 | 3.81E+05 | 1.50E+06 | 1.01E+06 | 7.70E+05 |
| Lmdn001560 | 6-DeoxyCatalpol | C15H22O9 | Monoterpenoids | 4.24E+05 | 5.64E+05 | 7.81E+05 | 2.04E+05 | 2.76E+05 |
| Qazn002817 | Gardenoside-glucoside | C23H34O16 | Monoterpenoids | 6.90E+05 | 3.91E+05 | 5.00E+04 | 2.23E+05 | 6.68E+04 |
| Zbbn004440 | 10-O-Caffeoyl-geniposidic acid | C25H28O13 | Monoterpenoids | 4.64E+05 | 1.93E+05 | 5.65E+04 | 9.04E+04 | 3.65E+05 |
| Wasn002690 | Villosolside | C16H26O9 | Monoterpenoids | 4.12E+04 | 3.61E+04 | 2.04E+05 | 2.69E+05 | 3.19E+05 |
| Lmdn004314 | 6-O-p-Coumaroylajugol | C24H30O11 | Monoterpenoids | 8.80E+04 | 6.41E+04 | 8.31E+04 | 4.13E+04 | 1.93E+05 |
| Lalp004159 | Plantarenaloside | C16H24O9 | Monoterpenoids | 6.77E+04 | 7.99E+04 | 7.77E+04 | 8.26E+04 | 8.30E+04 |
| Qazp003214 | Genameside D | C23H34O15 | Monoterpenoids | 4.02E+04 | 3.09E+04 | 1.36E+05 | 5.55E+04 | 2.53E+04 |

**Table S14** Candidate genes of LAMT, OAT and CYP71 during post-modification of iridoids

| **gene** | **enzyme** | **Root** | **Stem** | **Leaf** | **Flower** | **Fruit** |
| --- | --- | --- | --- | --- | --- | --- |
| Hd_18862 | LAMT | 0.252558 | 0.605782 | 1.624984 | 0.924183 | 0.63694 |
| Hd_18413 | LAMT | 0.311521 | 0.118034 | 0.004414 | 0.015482 | 0.002373 |
| Hd_33257 | LAMT | 0.455651 | 0.38809 | 0.036386 | 0.105011 | 0.143825 |
| Hd_14131 | LAMT | 1.537087 | 0.377853 | 0.006428 | 0.040959 | 0.002145 |
| Hd_43703 | LAMT | 0.129094 | 0.061504 | 0.011438 | 0.001537 | 0 |
| Hd_07967 | LAMT | 0.307532 | 0.265002 | 0.093951 | 0.016002 | 0.02683 |
| Hd_03900 | LAMT | 0 | 0.005282 | 0.062848 | 0 | 0 |
| Hd_25809 | LAMT | 0 | 0.013499 | 0.420606 | 0.032175 | 0.004477 |
| Hd_08117 | LAMT | 1.577419 | 2.07179 | 2.582865 | 0.274903 | 0.243258 |
| Hd_40849 | LAMT | 11.86302 | 5.498514 | 3.031279 | 0.710492 | 0.584708 |
| Hd_23009 | LAMT | 0.003832 | 0 | 0.056052 | 0 | 0 |
| Hd_32268 | LAMT | 16.27919 | 14.28628 | 13.17926 | 11.78425 | 6.824376 |
| Hd_30399 | OAT | 21.86165 | 17.43572 | 16.59199 | 16.45845 | 19.94648 |
| Hd_17223 | OAT | 6.02363 | 4.767692 | 3.596903 | 4.985043 | 7.561196 |
| Hd_20694 | OAT | 0.108549 | 0.015565 | 0.034752 | 0.017651 | 0.08584 |
| Hd_07742 | OAT | 0.00452 | 0 | 0 | 0 | 0.007986 |
| Hd_18362 | OAT | 392.713 | 190.71 | 85.97058 | 57.99698 | 40.27415 |
| Hd_33633 | OAT | 5.44738 | 6.042821 | 10.42838 | 7.315458 | 25.33856 |
| Hd_22877 | OAT | 96.59855 | 10.01112 | 0.094447 | 8.908248 | 14.4598 |
| Hd_04820 | OAT | 0.197609 | 3.56867 | 0.569719 | 0.438357 | 6.629102 |
| Hd_44929 | OAT | 0.11559 | 1.535336 | 0.376736 | 4.956749 | 6.604214 |
| Hd_41896 | OAT | 41.175 | 17.23111 | 21.29377 | 3.772481 | 6.172238 |
| Hd_43647 | OAT | 1.224716 | 2.787006 | 2.630229 | 3.595698 | 5.727199 |
| Hd_08909 | OAT | 57.10479 | 17.41635 | 13.8227 | 7.7568 | 4.796081 |
| Hd_20704 | OAT | 0.160744 | 0.345356 | 0.950738 | 0.268087 | 2.396136 |
| Hd_08777 | OAT | 5.61594 | 1.532636 | 4.024935 | 3.53815 | 2.203913 |
| Hd_08887 | OAT | 0.103449 | 0.170015 | 0.467417 | 0.45371 | 1.754637 |
| Hd_33631 | OAT | 0 | 0.026261 | 0.019115 | 0.187225 | 1.711395 |
| Hd_44928 | OAT | 8.715742 | 1.046407 | 1.693254 | 1.23991 | 0.355998 |
| Hd_08888 | OAT | 0 | 0.010011 | 0.044127 | 0 | 0.266758 |
| Hd_26006 | OAT | 0.005182 | 0.006705 | 0 | 0 | 0.020482 |
| Hd_26009 | OAT | 0.24316 | 0.00711 | 0.01131 | 0.012137 | 0.002117 |
| Hd_02203 | CYP71D55 | 0.274783 | 0.333692 | 1.879807 | 0.06889 | 0.090822 |
| Hd_07584 | CYP71D55 | 1.432706 | 1.730516 | 3.57782 | 0.517247 | 0.519202 |
| Hd_07586 | CYP71D55 | 0.252558 | 0.605782 | 1.624984 | 0.924183 | 0.63694 |
| Hd_07970 | CYP71D55 | 0.311521 | 0.118034 | 0.004414 | 0.015482 | 0.002373 |
| Hd_07973 | CYP71D55 | 0.455651 | 0.38809 | 0.036386 | 0.105011 | 0.143825 |
| Hd_11653 | CYP71D55 | 1.537087 | 0.377853 | 0.006428 | 0.040959 | 0.002145 |
| Hd_15702 | CYP71D55 | 0.129094 | 0.061504 | 0.011438 | 0.001537 | 0 |
| Hd_18015 | CYP71BE52 | 0.307532 | 0.265002 | 0.093951 | 0.016002 | 0.02683 |
| Hd_18118 | CYP71D55 | 0 | 0.005282 | 0.062848 | 0 | 0 |
| Hd_18119 | CYP71D55 | 0 | 0.013499 | 0.420606 | 0.032175 | 0.004477 |
| Hd_18364 | CYP71BE52 | 1.577419 | 2.07179 | 2.582865 | 0.274903 | 0.243258 |
| Hd_18368 | CYP71D55 | 11.86302 | 5.498514 | 3.031279 | 0.710492 | 0.584708 |
| Hd_25106 | CYP71D55 | 0.003832 | 0 | 0.056052 | 0 | 0 |
| Hd_27810 | CYP71D55 | 16.27919 | 14.28628 | 13.17926 | 11.78425 | 6.824376 |
| Hd_28527 | CYP71BE52 | 10.95368 | 8.139215 | 7.299835 | 6.138489 | 2.543671 |
| Hd_32361 | CYP71D55 | 0.717214 | 0.698134 | 0.046917 | 0.012103 | 0.559863 |
| Hd_32362 | CYP71BE52 | 109.8717 | 57.81626 | 6.117536 | 1.057353 | 1.909001 |
| Hd_35963 | CYP71D55 | 0.184653 | 0.119396 | 0.464588 | 0.031556 | 0.044547 |
